# Male Sex, Early Postoperative C-Reactive Protein and Prolonged Hospital Stay After Lung Resection in Older Patients: A Retrospective Cohort Study

**DOI:** 10.3390/diseases14100369

**Published:** 2026-10-06

**Authors:** Yeon Soo Kim, So Hyun Ahn

**Affiliations:** 1 College of Life Sciences and Biotechnology, Korea University, 145 Anam-ro, Seongbuk-gu, Seoul 02841, Republic of Korea; dustn0502@korea.ac.kr; 2 Ewha Medical Research Institute, School of Medicine, Ewha Womans University, Seoul 03760, Republic of Korea; 3 Ewha Medical Artificial Intelligence Center, School of Medicine, Ewha Womans University, Seoul 03760, Republic of Korea

**Keywords:** aged, sex characteristics, C-reactive protein, thoracic surgery, length of stay, pneumonectomy

## Abstract

Background/Objectives: Older patients make up a growing share of the lung-resection workload, and C-reactive protein measured in the first days after operation is used to judge recovery. Whether the same concentration means the same thing in older men and older women is not established. Methods: We analysed 724 lung resections in 716 patients from VitalDB, an open perioperative dataset from a Korean tertiary hospital; 313 (43.2%) were aged 65 years or older. The primary outcome was a postoperative stay of 7 days or longer. Older general surgical patients from the same institution served as an age-matched negative control. Results: Among patients aged 65 or older, prolonged stay occurred in 55 of 192 men (28.6%) and 10 of 121 women (8.3%), adjusted odds ratio 3.031 (95% CI 1.425–6.446, *p* = 0.0040). Median day 1 C-reactive protein was 51.9 mg/L in older men and 27.4 mg/L in older women, adjusted ratio of geometric means 1.825 (1.593–2.090, *p* = 1.8 × 10^−16^). C-reactive protein predicted prolonged stay unadjusted (1.594 per doubling, 1.292–1.967) but not once sex entered the model (0.865, 0.691–1.084, *p* = 0.208), an interval that remains compatible with a modest association in either direction. In 1635 older general surgical patients, neither effect was present (0.863, *p* = 0.302; 1.238, *p* = 0.163). The sex-by-age interaction was not significant (*p* = 0.519). Conclusions: In older patients undergoing lung resection, men mount a larger early inflammatory response than women to comparable operations and are three times as likely to stay a week or longer, an association absent from older general surgical patients at the same institution. In this dataset and with the measured covariates, C-reactive protein on the first postoperative day was not independently associated with prolonged hospital stay after adjustment for sex and the other available factors; with 113 events, this does not establish absence of prognostic value. These data do not derive, validate, or justify sex-specific C-reactive protein thresholds, and none is proposed.

## 1. Introduction

Lung resection is among the most common major thoracic operations, and an increasing proportion of it is performed in older patients. Perioperative care has been reorganised around enhanced recovery pathways that aim to return patients home within a few days [1,2], and length of postoperative stay has become the practical currency of that effort: recorded for every patient, integrating the events that matter, and used as an institutional quality metric after uncomplicated lobectomy [3,4,5]. Identifying in the first days which patients will not follow the expected trajectory is a concrete clinical problem, and it is sharpest in the old. Ageing brings a chronic low-grade elevation of circulating inflammatory mediators, commonly termed inflammageing, which raises the baseline against which any postoperative rise must be judged, and it brings reduced physiological reserve, so that a given insult is less well tolerated [6,7]. The older patient is therefore both the one in whom the marker is hardest to interpret and the one in whom being wrong costs most.

C-reactive protein is the marker most often used for that purpose: a hepatically synthesised acute-phase reactant responding to interleukin-6 over a wide dynamic range and measured almost universally after major surgery [8]. Its postoperative kinetics are well characterised—concentrations rise from the end of operation, peak on the second or third day and then fall—and a failure to fall has been proposed as an early signal of infection across abdominal, cardiac, orthopaedic and oesophageal surgery [9,10,11,12,13]. The size of the rise tracks the size of the tissue injury, as shown by the smaller responses to laparoscopic than to open cholecystectomy [14,15]. In thoracic surgery, early trajectories have been examined only on a small scale: the most comparable study analysed 144 patients, found that higher peaks with open surgery and more extensive resection were observed but not statistically confirmed, and called for prospective work to validate thresholds [16].

That postoperative C-reactive protein differs between men and women is not itself a new observation, but it has been made almost entirely in orthopaedic surgery. The postoperative course of the marker after total knee arthroplasty has been described explicitly as sex-specific [17], and comparable differences have been reported after total hip and shoulder arthroplasty [18,19,20]. Those studies established that the difference exists; none asked what it does to the prognostic use of the marker, and none examined thoracic surgery, where the marker is used differently and where the outcome at stake is recovery from a resection of functioning lung.

A second body of literature, largely unconnected to either, documents that the inflammatory response itself differs by sex. Sex hormones act on innate immune cells through oestrogen and androgen receptors, interact with glucocorticoid signalling, and the lung shows sex-dependent immune behaviour of its own [21,22,23,24]; longitudinal profiling during gender-affirming testosterone treatment shows the innate compartment shifting within months [25]. Circulating C-reactive protein differs between the sexes in the general population, sufficiently that sex-specific reference intervals have been derived and that its relationship with mortality is itself sex-dependent [26,27].

That the two exist separately matters, because male sex is already an established risk factor after lung resection: men experience more postoperative pulmonary complications, a difference that has been attributed to biological factors rather than to case mix [28,29,30,31,32], although no study has demonstrated the mechanism. Sex differences in perioperative outcome are reported after cardiac, hepatic, and lobectomy surgery [33,34,35], and the lung cancer population from which most resections are drawn differs between the sexes in histology, stage, and survival [36,37]. If sex is associated with both how much C-reactive protein rises and how well the patient does, an association between marker and outcome may in part reflect the absence of adjustment for sex. Precedent exists: sex-specific decision limits for cardiac troponin are established practice, and sex-specific interpretation of preoperative haemoglobin changes perioperative risk estimates [38,39,40]. Failure to analyse by sex remains a recognised weakness of this evidence base [41].

We therefore examined, in a cohort of lung resections from a public perioperative dataset and with particular attention to patients aged 65 years and older, whether C-reactive protein on the first postoperative day differs between men and women after comparable operations; whether prolonged postoperative stay differs by sex; and whether any association between the marker and prolonged stay survives adjustment for sex. To test whether a finding was specific to thoracic surgery rather than a property of the institution, its laboratory, or its population, we repeated the analysis in general surgical cases from the same dataset as an internal negative control [42,43].

## 2. Materials and Methods

### 2.1. Study Design and Data Source

This was a retrospective cohort study reported in accordance with the Strengthening the Reporting of Observational Studies in Epidemiology recommendations [44]. Data were obtained from VitalDB, an open perioperative dataset of 6388 non-cardiac surgical cases from ten operating theatres at Seoul National University Hospital, Republic of Korea [45], which combines intraoperative biosignals with electronic record data on demographics, physical status, operative details, laboratory results, and in-hospital outcomes, and has been used for secondary analyses of perioperative risk [46,47,48]. Critically for the present question, its laboratory table is longitudinal and includes postoperative as well as preoperative results.

The clinical information and laboratory tables were downloaded from the public application programming interface and are distributed under a Creative Commons Attribution-NonCommercial-ShareAlike 4.0 International licence. All analyses used the deidentified public release; no attempt was made to reidentify individuals, and no additional data were obtained from the source institution. The two tables were retrieved on 20 August 2026 from https://api.vitaldb.net/cases (accessed on 20 August 2026) (accessed on 20 August 2026) and https://api.vitaldb.net/labs (accessed on 20 August 2026) (accessed on 20 August 2026); the files analysed carry MD5 checksums 427dafa2a716dd9f98b09f7de08b0a0e and 811c556f9354b3fd0c62e3770c455553 respectively, and the cohort-construction script prints both so that the exact version analysed can be identified. The source data were collected at Seoul National University Hospital between August 2016 and June 2017 [45].

### 2.2. Participants

Cases were eligible if performed by the thoracic surgery service, recorded as a major or minor resection, and carrying an operation name denoting resection of lung parenchyma: lobectomy, bilobectomy, sleeve lobectomy, pneumonectomy, segmentectomy, wedge resection, or metastasectomy (Table 1). Of 1111 thoracic cases, 827 were major or minor resections, and 758 carried a qualifying operation name; 69 were excluded because the recorded operation was a biopsy (34), bullectomy (30), empyemectomy (3), or unspecified excision (2). Cases then required a C-reactive protein concentration both within 30 days before the case and on the first postoperative day, leaving 724 cases in 716 patients. The flow of cases from the source dataset to the analytical cohort, with the reason and number for each exclusion, is given in Figure S1. No age restriction was applied during cohort construction, so the cohort represents the resection workload as performed; 313 cases (43.2%) were in patients aged 65 years or older, and this group is the focus of the present report. Results are reported per surgical case, with the number of patients alongside. The 724 cases arose in 716 patients: 708 patients contributed one operation, and eight contributed two. Every timestamp in the source dataset is expressed in seconds from the start of the case to which it belongs, and no calendar date is released, so neither the interval between two operations in the same patient nor their order in time can be recovered. The interval from admission to discharge is independent of that anchor, and it differed between the two operations in six of the eight patients, which establishes that those pairs belonged to separate admissions; in three of the eight, the recorded age also differed by one year, placing the operations at least months apart. In the remaining two patients, the two admissions were of equal recorded length and cannot be distinguished. Analyses treating each operation as an independent observation are therefore reported alongside an analysis restricted to one operation per patient and two analyses that allow for clustering within patients, which are given with the sensitivity analyses.

### 2.3. Exposures and Outcome

The principal exposure was sex as recorded. The marker examined was C-reactive protein on the first postoperative day. Postoperative windows were assigned from the start of the case, which is the origin of the time axis in the source dataset: the day 1 value is the last result more than 0 and up to 1.5 days after the start of the case, and the day 3 value is the last result more than 2.5 and up to 4 days after it; the preoperative value is the last within 30 days before the case. Referred instead to the end of operation, which is what the clinician sees, the day 1 samples were drawn a median of 18.0 h afterwards (range 0.3 to 34.1) and the day 3 samples a median of 69.7 h afterwards (57.5 to 93.2); no day 1 or day 3 sample preceded the end of operation. Where more than one result fell inside a window, which occurred in 682 cases on day 1 and 103 on day 3, the last was taken. Concentrations are reported in mg/L. The definition and source field of every variable used in the analysis are set out in Table S1. C-reactive protein was markedly right-skewed and analysed after base-two logarithmic transformation, so coefficients express change per doubling; for the sex comparison, they are back-transformed and reported as ratios of geometric means, the multiple by which concentrations in men exceed those in women.

The primary outcome was a postoperative stay of 7 days or longer, from the end of operation to discharge. The threshold was set against published practice rather than against the distribution in this cohort. In 13,099 lobectomies from which complications and deaths had been excluded, the median stay was 5 days with an interquartile range of 4 to 7, so a stay of 7 days or longer lies at the upper limit of what an explicitly uncomplicated recovery produces [3]; the Society of Thoracic Surgeons risk model for the same operation places its own threshold at 14 days in a population whose whole distribution is longer [4]; and seven days exceeds the target of the enhanced recovery pathways against which this workload is managed [1,2]. Median stay in the present cohort was 3.6 days, so the threshold identifies the upper tail while retaining 113 events. The outcome is length of hospital stay and is named as such throughout: it is not a measure of postoperative morbidity, and no statement about complications is made from it. Critical care admission was deliberately not used: 400 cases (55.2%) entered critical care but only 15 (2.1%) stayed three days or longer, indicating that most admissions were planned on the basis of the scheduled operation rather than triggered by deterioration, a well-described problem in such analyses [49]. In-hospital death occurred in four cases and could not be analysed.

### 2.4. Covariates

Adjusted models in the whole cohort included age in decades, body mass index, American Society of Anesthesiologists physical status dichotomised at grade III or above [50], emergency operation, extent of resection, open thoracotomy versus a minimally invasive approach, and duration of surgery in hours. Within the older subgroup the number of events does not support nine parameters, so models there used a reduced set of five covariates—age, physical status, extent of resection, open approach and duration of surgery—which keeps the ratio of events to parameters above the conventional minimum of ten [51]; estimates from the full covariate set are reported alongside so that the effect of the reduction can be seen. Extent and duration were included deliberately: because the size of the inflammatory response tracks the size of the operation [14,52,53,54], an unadjusted sex difference could simply reflect men receiving larger operations, and only adjustment for what was actually done distinguishes the two. Two further recorded fields bear on the airway and oncological factors that are otherwise absent. The dataset releases an interpretation of preoperative spirometry for every case and a preoperative diagnosis from which the indication for resection can be classified. Neither entered the primary models, which were fixed before these analyses; both are used in an additional analysis added at revision and reported with the results. Neither is a substitute for smoking history, for a formal diagnosis of chronic obstructive pulmonary disease, or for pathological stage, none of which the dataset releases.

### 2.5. Negative Control

The general surgical cases were used as a negative control population, both in their entirety and restricted to patients aged 65 years or older, so that the comparison is matched for age: a setting in which the same exposure, outcome, and models are applied where the hypothesised effect is not expected, so that an effect appearing in both would indicate shared confounding or measurement artefact rather than a specialty-specific phenomenon [42,43]. The same models were fitted in the 4930 general surgical cases, replacing extent of resection with the recorded operation category (nine groups) and leaving all other covariates identical. These cases were operated at the same institution, in the same period, with results from the same laboratory, so a difference between the settings cannot be attributed to assay, population or era.

### 2.6. Statistical Analysis

Continuous variables are summarised as median with interquartile range and compared with the Mann–Whitney U test; categorical variables as counts with percentages, compared with the chi-squared test or Fisher’s exact test where any expected count was below five. The sex difference in C-reactive protein was estimated by linear regression on the base-two logarithm of the concentration, and associations with prolonged stay by logistic regression reported as odds ratios with 95% confidence intervals. To locate where the association between marker and outcome changed, six nested models were fitted to the same cases in a prespecified order, and the two variables introduced together at the third step were then entered separately. Analyses were also stratified by sex and repeated within age strata; because a difference between two subgroup estimates is not evidence of a difference in effect, a sex-by-age interaction was fitted, with age entered both as a binary variable at 65 years and as a continuous variable.

With 113 events and nine parameters, the ratio of events to parameters exceeded the conventional minimum of ten [51]. Discrimination, calibration by the Hosmer–Lemeshow test, and the ratio of events to parameters for each model are reported in Table S3, and variance inflation factors for the covariates of the full model are in Table S4. Missing data were confined to physical status (20 cases, 2.8%), albumin (one), and white cell count (two); physical status was carried into the models with a missing indicator rather than discarding records [55]. Two cases carried a recorded physical status of grade VI, presumed a coding error, and both were retained so the cohort matched its prespecified definition. Exact *p* values are reported throughout. Analyses used Python 3.11.10 with pandas 2.3.3, numpy 2.4.6, statsmodels 0.15.0, scipy 1.17.1 and matplotlib 3.11.1; two-sided *p* values below 0.05 were considered significant.

Because a logistic model constrains each covariate to act additively on the log-odds scale, the absence of an independent association with C-reactive protein could in principle be an artefact of that form. The analysis was therefore repeated across thirteen model classes and four variable sets, with discrimination estimated by repeated stratified five-fold cross-validation, and nested curves were compared by the method of DeLong et al. for correlated curves [56]. The model classes, the software used, and the accompanying permutation-importance, Shapley, partial-coefficient-of-determination, sex-stratified and decision-curve analyses are specified in the Supplementary Methods. They address model form and structure, not causation. The causal structure assumed throughout, and the distinction between biological sex as an exposure and the unmeasured clinical factors associated with it, is set out at the start of Section 4.

Seven sensitivity analyses tested whether the principal estimates depended on the cohort definition or on the assumption that operations are independent: one operation per patient; cluster-robust standard errors with the patient as the clustering unit; generalised estimating equations with an exchangeable working correlation and the patient as the group; a stay of 10 days or longer as the outcome; complete-case analysis of physical status; exclusion of the two grade VI cases; and addition of the preoperative concentration to the adjusted models. The one-operation-per-patient analysis retains the operation with the lower case identifier. Case identifiers in the source dataset are random numbers and carry no temporal information, so this selects one operation per patient at random with respect to time; it is not, and is not described as, the first operation in time. Analyses by age stratum and by age cut-off are reported separately. The missing indicator for physical status proved perfectly predictive: none of the 20 cases with a missing grade had a prolonged stay, which produces separation in the logistic model; the complete-case analysis therefore serves as the check on that covariate, and the indicator coefficient is not interpretable.

An analysis of the trajectory between day 1 and day 3 was also performed and is reported as exploratory. It was undertaken after several combinations of exposure and timepoint had already been examined in the same data; no correction for multiple comparisons has been applied, and it is not offered as a confirmatory result.

Two points of reporting transparency should be stated. The cohort definition and the primary analysis were fixed before the analyses reported here, and reside in a single script. The decision to bring the older subgroup to the front was taken after the age-stratified analyses had been run; the age strata are therefore presented as a subgroup analysis of a cohort assembled without an age restriction, and the formal interaction test is reported whether or not it supports the emphasis.

### 2.7. Ethical Considerations

This is a secondary analysis of a publicly available, deidentified dataset involving no new contact with human participants; it falls outside the remit of institutional ethics review and qualifies for exemption. Collection and public release of the source dataset were approved by the Institutional Review Board of Seoul National University Hospital (approval number H-1408-101-605) and registered at ClinicalTrials.gov (NCT02914444); written informed consent was waived by that board because the released data are anonymous [45].

## 3. Results

### 3.1. Cohort

Of 1111 thoracic cases, 827 were major or minor resections, and 758 carried a qualifying operation name; 724 of these, in 716 patients, had C-reactive protein measured both before operation and on the first postoperative day and formed the analytical cohort (Figure S1; Table 1). Median age was 62 years (interquartile range 55 to 70), and 313 cases (43.2%) were in patients aged 65 years or older, of whom 192 were men and 121 women. There were 381 men and 343 women in the cohort as a whole. Major resection was performed in 347 cases (47.9%), open thoracotomy in 46 (6.4%), and 13 operations (1.8%) were emergencies. Median duration of surgery was 2.07 h, and median postoperative stay was 3.6 days (2.4 to 5.5); 113 cases (15.6%) stayed 7 days or longer and 56 (7.7%) 10 days or longer. Day 3 C-reactive protein was available in 394 cases (54.4%).

Men and women differed across the operative variables in the direction expected to produce a spurious inflammatory difference. Men were older (median 65 versus 61 years, *p* = 6.0 × 10^−6^), more often of physical status grade III or above (14.3% versus 5.1%, *p* = 4.8 × 10^−5^), more often underwent major resection (52.5% versus 42.9%, *p* = 0.0096) and open thoracotomy (10.0% versus 2.3%, *p* = 2.6 × 10^−5^), and had longer operations (2.25 versus 1.83 h, *p* = 1.2 × 10^−11^); preoperative C-reactive protein was also higher (1.20 versus 0.60 mg/L, *p* = 2.5 × 10^−8^). Any analysis of postoperative inflammation by sex must therefore account for the operation performed.

### 3.2. Sex and the Early Inflammatory Response

**Figure 1:**
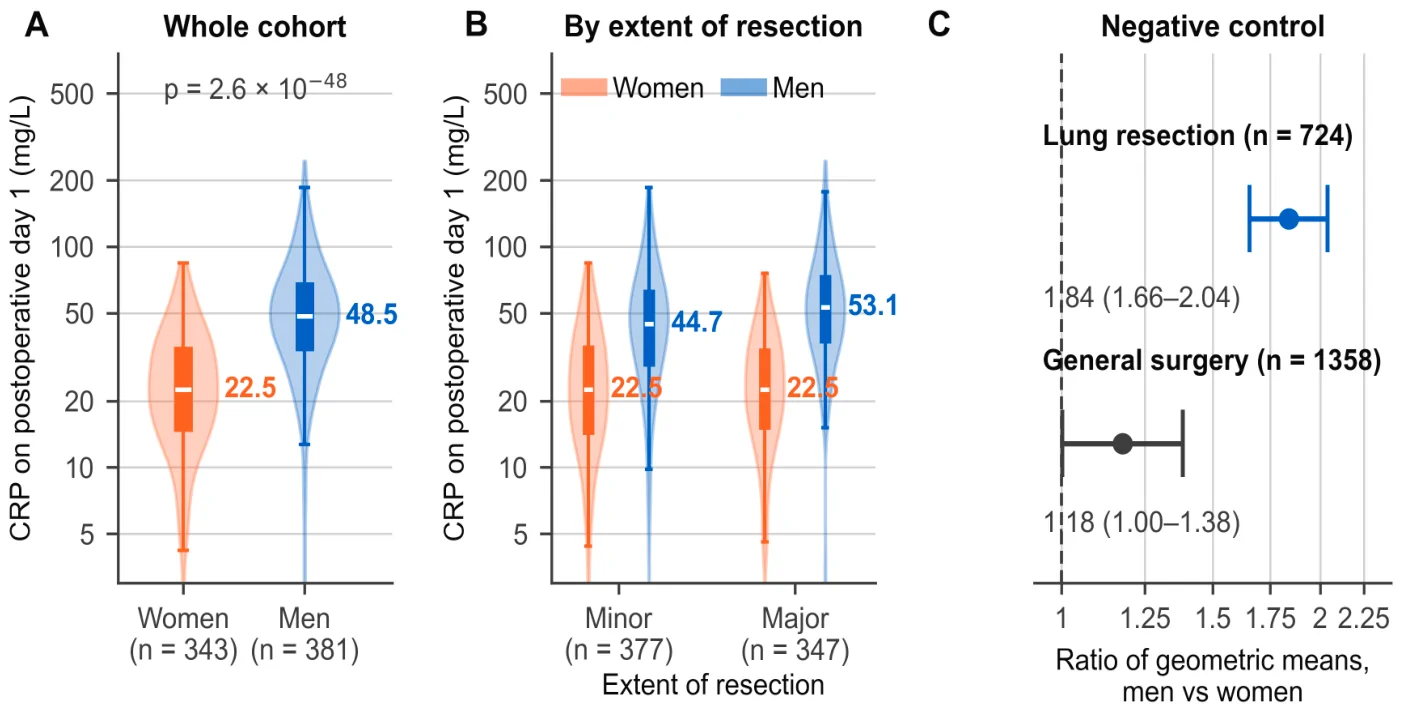
Sex differences in C-reactive protein on the first postoperative day after lung resection. Panel (**A**), distribution in women and men; violins show the full distribution, boxes the interquartile range and the white line the median, and the *p* value is from a Mann–Whitney U test. Panel (**B**), the same comparison within minor and within major resections. Panel (**C**), ratio of geometric means for men relative to women, adjusted as described in Table 2, in the lung-resection cohort and in general surgical cases at the same institution over the same period; horizontal lines are 95% confidence intervals, and the broken line marks a ratio of one. Vertical axes in panels (**A**,**B**) are logarithmic.

C-reactive protein on the first postoperative day was 48.5 mg/L in men and 22.5 mg/L in women (medians; *p* = 2.6 × 10^−48^), a ratio of geometric means of 2.140 (95% confidence interval 1.924 to 2.379, *p* = 7.3 × 10^−40^) before adjustment (Table 2, panel A; Figure 1A). Adjustment for age, body mass index, physical status, urgency, extent of resection, approach, and duration reduced this only slightly, to 1.838 (1.656 to 2.040, *p* = 5.3 × 10^−28^), so the difference was not explained by men receiving larger operations.

The pattern held within strata of operative extent (Figure 1B): median C-reactive protein in women was essentially identical after minor and after major resection (22.5 and 22.5 mg/L), whereas in men it rose with extent (44.7 and 53.1 mg/L). The response in women was almost insensitive to how much lung was removed over the range of operations performed here, while in men it was both higher and graded by extent.

### 3.3. Sex and Prolonged Stay

A stay of 7 days or longer occurred in 88 of 381 men (23.1%) and 25 of 343 women (7.3%), an unadjusted odds ratio of 3.820 (2.384 to 6.123, *p* = 2.6 × 10^−8^) and an adjusted odds ratio of 2.529 (1.503 to 4.254, *p* = 0.00047) (Table 2, panel B). Adding day 1 C-reactive protein did not attenuate the effect of sex but increased it slightly, to 2.874 (1.644 to 5.026, *p* = 0.00021); the inflammatory response therefore does not act as an intermediate step between sex and prolonged stay in these data.

### 3.4. C-Reactive Protein and Prolonged Stay

Taken alone, C-reactive protein on day 1 predicted prolonged stay, odds ratio 1.594 per doubling (1.292 to 1.967, *p* = 1.3 × 10^−5^); in the fully adjusted model including sex, it was 0.865 (0.691 to 1.084, *p* = 0.208) (Table 2, panel C). Stratified analysis showed no association within either sex: 0.867 in men (0.653 to 1.151, *p* = 0.324) and 0.873 in women (0.594 to 1.283, *p* = 0.490).

**Figure 2:**
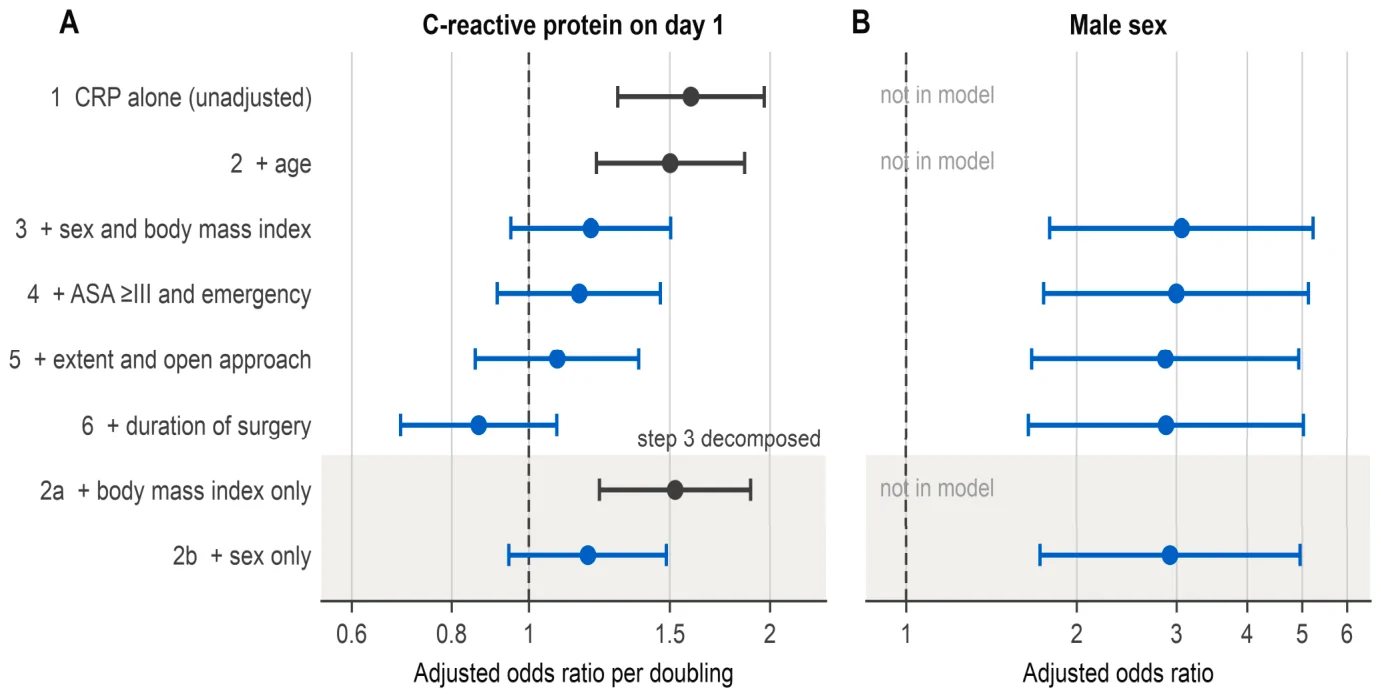
Where the association between C-reactive protein and prolonged stay disappears. Adjusted odds ratios for a stay of 7 days or longer from six nested logistic models fitted to the same 724 cases with 113 events, each adding the variables named to those above. Panel (**A**), C-reactive protein on day 1, per doubling. Panel (**B**), male sex, in the models containing it. Points are odds ratios, horizontal lines 95% confidence intervals, the broken line an odds ratio of one; both axes are logarithmic. The shaded band separates the two variables introduced at the third step: body mass index alone leaves the C-reactive protein estimate intact, whereas sex alone accounts for the whole change.

Table 3 and Figure 2 locate where the association disappears. Adjustment for age alone left it intact (1.502, *p* = 1.8 × 10^−4^); when sex and body mass index were added together at the third step, it fell to 1.195 (0.949 to 1.505, *p* = 0.129) and did not recover with any later covariate. Entering the two separately identified sex as responsible: with body mass index alone the estimate remained 1.523 (1.225 to 1.894, *p* = 1.5 × 10^−4^), whereas with sex alone it fell to 1.185 (0.944 to 1.486, *p* = 0.143). Across the same sequence the estimate for male sex was essentially unchanged, from 3.062 to 2.874.

### 3.5. Internal Negative Control

In the 4930 general surgical cases from the same institution and period, neither effect was present (Table 2, panel D; Figure 1C). The adjusted ratio of geometric means was 1.178 (1.002 to 1.385, *p* = 0.048) in the 1358 cases with a day 1 measurement, against 1.838 after lung resection, and male sex was not associated with prolonged stay (1.131, 0.952 to 1.344, *p* = 0.161) despite 1479 events, against 2.529 after lung resection. The absence of an effect in general surgery is therefore not a matter of statistical power.

### 3.6. Older Patients

Among the 313 cases in patients aged 65 years or older, a postoperative stay of 7 days or longer occurred in 55 of 192 men (28.6%) and in 10 of 121 women (8.3%). The adjusted odds ratio for male sex was 3.031 (1.425 to 6.446, *p* = 0.0040) in the reduced model, and 3.240 (1.475 to 7.118, *p* = 0.0034) when the full covariate set was used (Table 4). The median C-reactive protein on the first postoperative day was 51.9 mg/L in older men and 27.4 mg/L in older women, with an adjusted ratio of geometric means of 1.825 (1.593 to 2.090, *p* = 1.8 × 10^−16^).

In the 411 cases among patients under 65, the corresponding estimates were 1.905 for male sex (0.943 to 3.850, *p* = 0.072) and 1.873 for the inflammatory difference (1.605 to 2.184, *p* = 1.2 × 10^−14^). The inflammatory difference was therefore the same at both ages, whereas the outcome estimate was larger in the older group and reached conventional significance only there.

**Figure 3:**
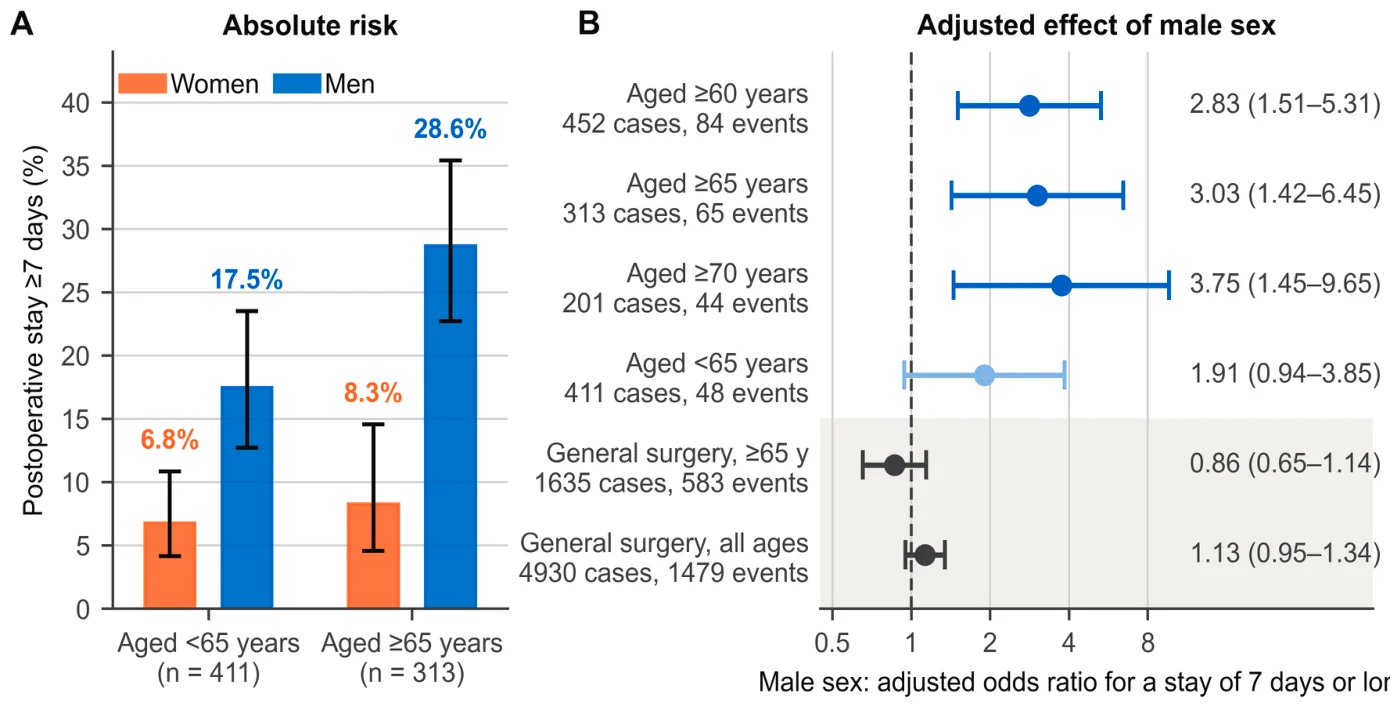
Prolonged postoperative stay by age and sex after lung resection. Panel (**A**), proportion of cases with a postoperative stay of 7 days or longer, by age group and sex, with 95% Wilson confidence intervals; the four groups contain 222 women and 189 men aged under 65 years and 121 women and 192 men aged 65 years or older. Panel (**B**), adjusted odds ratios for the same outcome in men relative to women, by age stratum and by specialty, with 95% confidence intervals; the broken line marks an odds ratio of one and the axis is logarithmic. Lung-resection strata use the reduced covariate set (age, physical status, extent of resection, open approach and duration of surgery); general surgical models replace extent of resection with operation category and retain the full covariate set. The shaded band separates the general surgical negative controls. Strata above 75 years contained too few events for a stable estimate and are not reported. The apparent gradient across age cut-offs is not supported by a formal sex-by-age interaction (*p* = 0.519).

That difference between strata must not be over-read. The formal interaction between sex and age was not significant, whether age was entered as a binary variable at 65 years (ratio of odds ratios 1.403, 0.501 to 3.929, *p* = 0.519) or as a continuous variable (1.188 per decade, 0.768 to 1.837, *p* = 0.439); the interaction with age for the inflammatory difference was likewise null (0.949, *p* = 0.615). The data are therefore compatible with an effect of the same size at all ages, and the confidence interval in the younger stratum includes the older estimate. What can be said without qualification is that the absolute risk is concentrated in the old: prolonged stay occurred in 20.8% of patients aged 65 or older versus 11.7% of those under 65. Among patients aged 65 or older, it affected 28.6% of men versus 8.3% of women, and among those under 65, 17.5% of men versus 6.8% of women (Figure 3A).

Across successive age cut-offs, the estimate for male sex moved monotonically upwards, from 2.83 (1.51 to 5.31) at 60 years and above, to 3.03 (1.42 to 6.45) at 65 and above, and to 3.75 (1.45 to 9.65) at 70 and above (Figure 3). Beyond 75 years, only 104 cases and 24 events remained and the model became unstable, so that stratum is not reported. The gradient is consistent in direction with the subgroup contrast but rests on progressively fewer events and, as above, is not supported by a significant interaction.

### 3.7. Age-Matched Negative Control

Restricting the general surgical comparison to the 1635 patients aged 65 years or older, with 583 prolonged stays, removed the effect entirely: the adjusted odds ratio for male sex was 0.863 (0.654 to 1.141, *p* = 0.302), and the adjusted ratio of geometric means for C-reactive protein on day 1 was 1.238 (0.917 to 1.672, *p* = 0.163) in the 466 older general surgical cases with a day 1 measurement (Table 4; Figure 3). Among older patients, therefore, the contrast between thoracic and general surgery is, if anything, sharper than in the cohorts taken as a whole.

### 3.8. Sensitivity Analyses

Table 5 reports seven sensitivity analyses, and Table S2 gives the complete set including every age stratum. Restricting the cohort to one operation per patient, which removes the eight repeated operations, left both estimates essentially unchanged: 1.851 (1.667 to 2.055) for the inflammatory difference and 2.634 (1.555 to 4.460, *p* = 0.00031) for male sex. Allowing for clustering within patients across all 724 operations gave 1.838 (1.649 to 2.049) and 2.529 (1.479 to 4.324, *p* = 0.00070) with cluster-robust standard errors, and 1.864 (1.671 to 2.080) and 2.538 (1.490 to 4.324, *p* = 0.00061) by generalised estimating equations; the association between C-reactive protein and prolonged stay with sex in the model remained null under both, 0.865 (0.713 to 1.050, *p* = 0.143) and 0.865 (0.714 to 1.049, *p* = 0.140). With eight repeated operations among 716 patients, the clustering is negligible, and these analyses confirm it rather than correct anything. The sex difference in C-reactive protein was otherwise stable across every definition, the adjusted ratio ranging from 1.741 to 1.864 with every *p* value below 10^−24^, and the association between male sex and prolonged stay ranged from 2.450 to 2.634, except with a stay of 10 days or longer as the outcome, which strengthened it to 4.457 (1.965 to 10.111, *p* = 0.00035). Because the missing-grade indicator was perfectly predictive of a non-prolonged stay, the complete-case analysis reproduced the primary estimate exactly.

### 3.9. Surgical Approach

Videoscopic surgery was used in 677 of the 724 operations (93.5%), open thoracotomy in 46, and a robotic approach in one. Within the videoscopic operations alone the sex difference in C-reactive protein was essentially that of the whole cohort, an adjusted ratio of geometric means of 1.820 (1.634 to 2.026, *p* = 1.1 × 10^−25^) against 1.838 overall, and male sex remained associated with prolonged stay, 2.834 (1.643 to 4.886, *p* = 0.00018) with 94 events (Table 6). Among the 290 videoscopic operations in patients aged 65 years or older, the corresponding estimates were 1.823 (1.588 to 2.093, *p* = 7.1 × 10^−16^) and 3.720 (1.636 to 8.458, *p* = 0.0017). The 46 open operations gave 1.603 (0.985 to 2.608, *p* = 0.057) with the same adjustment, and the formal interaction between sex and approach was null (ratio 0.931, *p* = 0.793). The difference is therefore not a property of the more invasive operations: it is present, and of the same size, in the minimally invasive practice that now predominates.

### 3.10. Airway Disease and Indication for Resection

An obstructive pattern on preoperative spirometry was recorded in 65 of the 724 cases (9.0%) and was five times as common in men as in women, 55 of 381 (14.4%) against 10 of 343 (2.9%), *p* = 1.3 × 10^−7^; a stay of 7 days or longer followed in 24.6% of those cases against 14.7% of the rest. The indication for resection also differed by sex: primary lung cancer accounted for 192 of the 381 operations in men (50.4%) and 109 of the 343 in women (31.8%). Both patterns run in the direction that would generate the findings of this report by confounding. Neither did so. Adding the obstructive pattern to the adjusted models moved the inflammatory difference from 1.838 to 1.876 (1.689 to 2.085) and the odds ratio for male sex from 2.529 to 2.558 (1.513 to 4.324); the full spirometry category gave 1.882 and 2.604, the indication gave 1.839 and 2.591, and both together gave 1.872 and 2.621 (Table 7). The association between C-reactive protein and prolonged stay with sex in the model remained null throughout, between 0.845 and 0.866. Recorded airway obstruction and the recorded indication therefore do not account for either finding. That is not the same as excluding smoking, which the dataset does not release and for which an interpretive spirometry category is at best a coarse proxy.

### 3.11. Flexible Models

**Figure 4:**
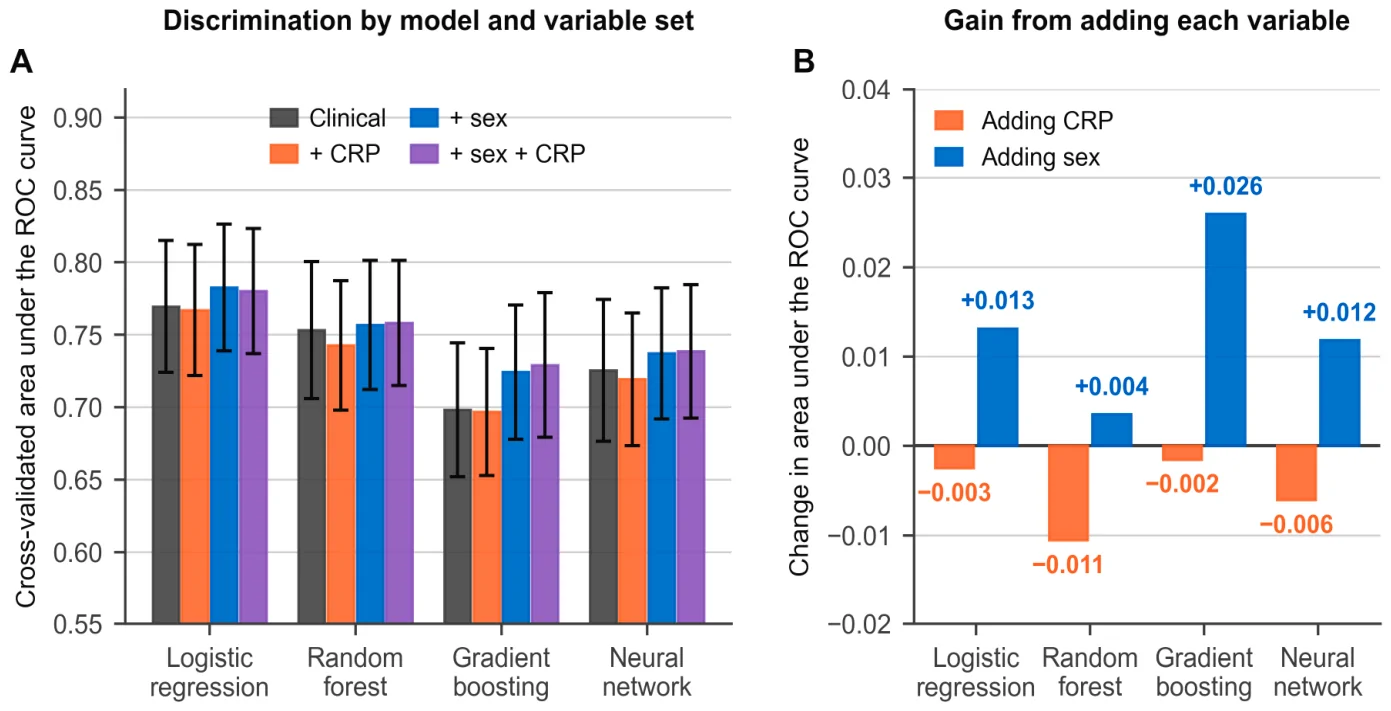
Discrimination of models with and without C-reactive protein and sex. Panel (**A**), area under the receiver operating characteristic curve from repeated stratified five-fold cross-validation with five repetitions, for four model classes and four variable sets; bars are means and whiskers one standard deviation across the 25 fits. Panel (**B**), the change in the area under the curve produced by adding C-reactive protein and by adding sex to the clinical covariates, for each model class. Adding the marker does not improve discrimination in any model, whereas adding sex does.

Across all four model classes, adding C-reactive protein to the clinical covariates did not improve discrimination, whereas adding sex did (Figure 4). The cross-validated area under the curve for the clinical covariates alone ranged from 0.698 to 0.769; adding C-reactive protein changed it by between −0.011 and −0.002, and adding sex changed it by between +0.004 and +0.026, so the direction was consistent across all four. The three flexible learners all discriminated less well than logistic regression, which is what would be expected with 724 cases and 113 events, and none extracted information from the marker that the simpler model had missed (Figure S2). The absence of an independent association between the marker and prolonged stay in this dataset is therefore not an artefact of the additive form of the logistic model, since three learners free of that constraint reach the same conclusion.

**Figure 5:**
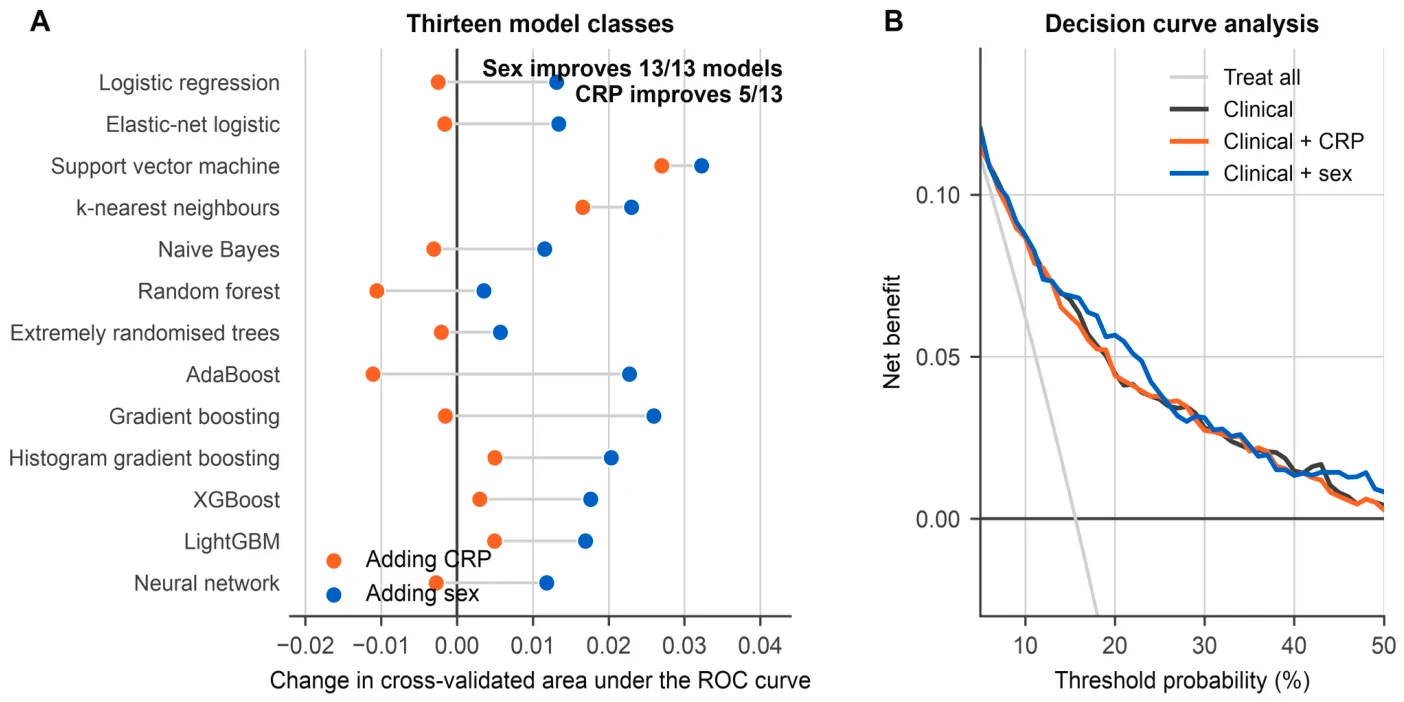
Thirteen model classes and clinical utility. Panel (**A**), the change in cross-validated area under the receiver operating characteristic curve produced by adding C-reactive protein and by adding sex to the clinical covariates, for each of thirteen model classes; the vertical line marks no change and the grey connector joins the two estimates for each model. Panel (**B**), decision curve analysis: net benefit across threshold probabilities for the clinical model, the same model with C-reactive protein, and with sex, together with the strategy of treating all patients. Adding the marker does not raise net benefit at any threshold.

Widening the comparison to thirteen model classes gave the same answer more emphatically (Figure 5A; Table S5 and Figure S4). Adding sex improved discrimination in all thirteen, by a median of 0.017; adding C-reactive protein improved it in five of thirteen, by a median of −0.002. The five in which the marker helped were the models that discriminated least well overall, and in none did the gain approach that from sex. Decision curve analysis agreed in clinical terms: across threshold probabilities from 5% to 50%, adding the marker never raised net benefit above the clinical model, whereas adding sex did over most of that range (Figure 5B).

Three of these analyses describe why. First, sex accounts for a large share of the marker itself: after the clinical covariates, the partial coefficient of determination for sex on day 1 C-reactive protein was 0.155, almost identical to that for duration of surgery (0.157) and nine times that for extent of resection (0.017) (Figure S5, panel C). Second, the relationship is confounding rather than effect modification: the sex-by-marker interaction was null (ratio of odds ratios 0.940, 0.596 to 1.481, *p* = 0.789). Third, within each sex, the marker added nothing, whether modelled additively or by a random forest. Shapley values ranked male sex above the marker, and the sex-by-marker interaction thirteenth of the forty-five variable pairs (Figure S5; Table S6).

Permutation importance requires a more careful reading and is reported in Figure S3. In the two tree-based models, the marker ranked above sex, whereas in logistic regression sex ranked above the marker—which would be expected if the two carry overlapping information, since a flexible model can use the marker as a proxy for the sex of the patient. Permutation importance measures what a fitted model relies on, not what determines the outcome, and should not be read as evidence that the marker is prognostic once sex is known.

### 3.12. Exploratory Analysis of the Trajectory

**Figure 6:**
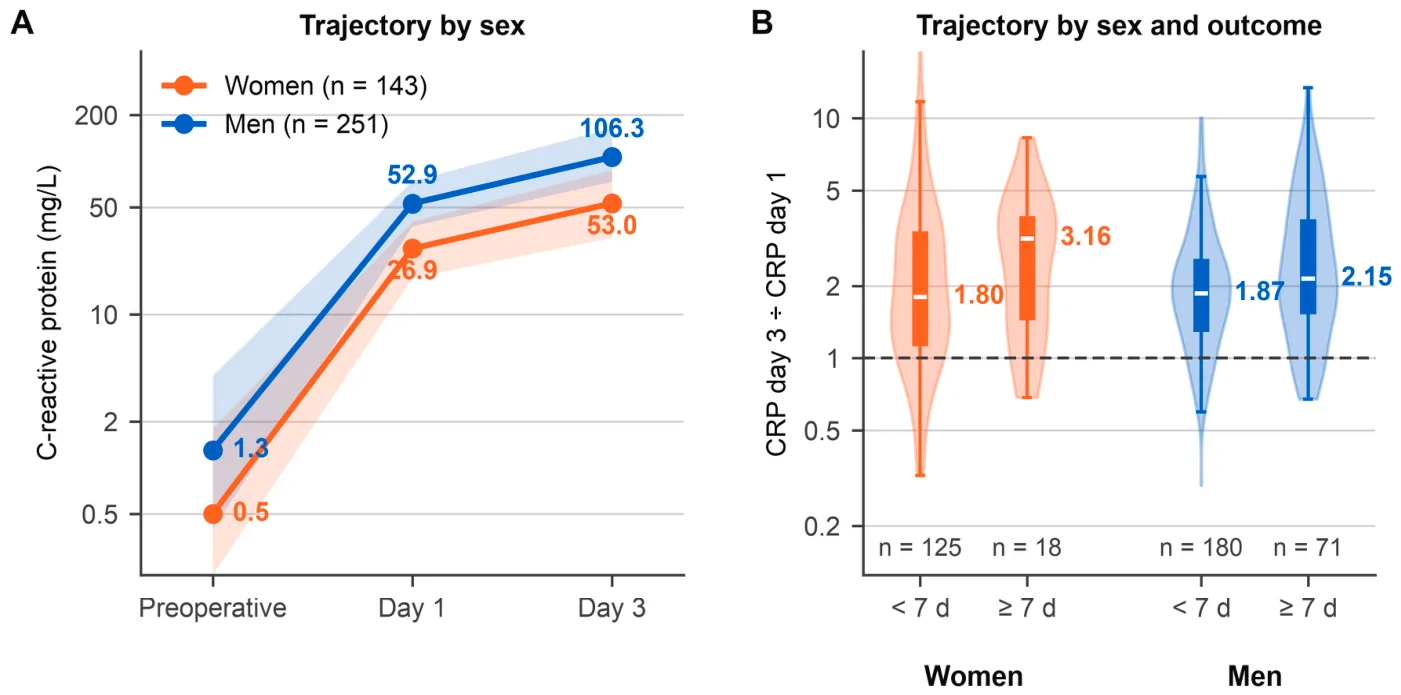
C-reactive protein trajectory by sex, and by sex and outcome. Panel (**A**), median concentration before operation and on postoperative days 1 and 3, shading showing the interquartile range, in the 394 cases with a day 3 measurement. Panel (**B**), the ratio of the day 3 to the day 1 concentration by sex and by whether stay reached 7 days; the broken line marks a ratio of one. Both vertical axes are logarithmic. Panel (**B**) is exploratory: the adjusted odds ratio followed examination of several exposure and timepoint combinations, no correction for multiple comparisons has been applied, and the subgroup of women staying 7 days or longer contains only 18 cases. d, days.

Among the 394 cases with a day 3 measurement, the ratio of day 3 to day 1 C-reactive protein was associated with prolonged stay after adjustment including sex (1.463 per doubling, 1.100 to 1.945, *p* = 0.0088), whereas the day 3 concentration itself was not (1.134, 0.824 to 1.560, *p* = 0.441) (Table 2, panel E; Figure 6). This analysis is exploratory: it followed examination of several exposure and timepoint combinations in the same data, no correction for multiple comparisons has been applied, and the subgroup of women with a prolonged stay contains only 18 cases. Table 8 separates the absolute concentrations from the ratio between them. Day 1 and day 3 concentrations were each about twice as high in men as in women, adjusted ratios of 1.824 (1.628 to 2.044) and 1.798 (1.580 to 2.046), but the ratio between the two days was the same in both sexes: 1.99 in men and 1.98 in women, an adjusted ratio of 0.985 (0.855 to 1.136, *p* = 0.839). The relative change between the two days is therefore the part of the trajectory that does not depend on the sex of the patient, while the absolute value on either day does. That observation must be weighed against how this subgroup arose. A day 3 measurement was available in 65.9% of men versus 41.7% of women, and in 78.8% of cases with a prolonged stay versus 49.9% of the rest, so the 394 are a strongly selected group; within them, even the day 1 estimate is distorted, reversing to 0.643 (0.451 to 0.916). It indicates a direction for prospective work, not a finding on which practice should change.

## 4. Discussion

In 724 lung resections, of which 313 were in patients aged 65 years or older, men had roughly twice the concentration of C-reactive protein on the first postoperative day that women had, a difference that persisted almost undiminished after adjustment for age, physical status, extent of resection, approach, and duration of surgery. Among the older patients, more than one man in four stayed in the hospital for a week or longer compared with one woman in twelve, an adjusted odds ratio of three. C-reactive protein appeared to predict prolonged stay until sex entered the model, at which point the association vanished and remained absent within each sex. In general surgical cases from the same institution, laboratory and period, neither the amplified response nor the outcome disadvantage was present.

**Figure 7:**
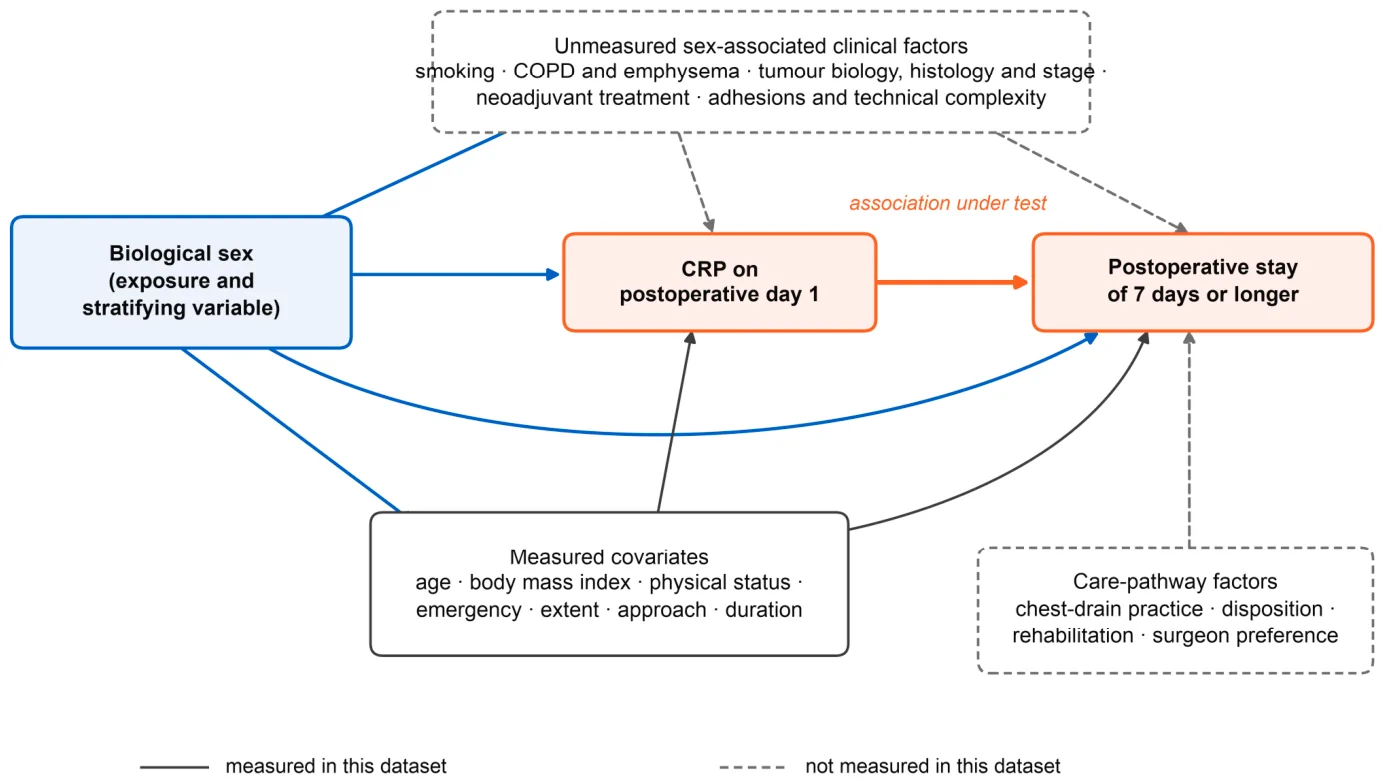
Assumed causal structure. Biological sex is shown as an exposure and stratifying characteristic rather than as a modifiable cause, distinguished from the unmeasured clinical factors associated with it—smoking, chronic obstructive pulmonary disease and emphysema, tumour biology, histology and stage, neoadjuvant treatment, and adhesions or technical complexity—which have paths to both the marker and the outcome. A separate group of unmeasured care-pathway factors, comprising chest-drain practice, discharge disposition, rehabilitation resource and surgeon preference, has a path to the outcome alone. Solid boxes and arrows denote variables measured in this dataset and broken variables that are not measured. Conditioning on sex closes the paths from sex but leaves those from the unmeasured factors open, so the attenuation of the association between C-reactive protein and prolonged stay after adjustment for sex is consistent with confounding but does not by itself establish it.

Before interpreting any of this, the causal structure being assumed should be stated, because the words confounding and mediation are used below in a restricted sense. Figure 7 sets it out. Biological sex is treated throughout as an exposure and a stratifying characteristic, not as a modifiable cause, and it is kept distinct from the clinical factors associated with it that this dataset does not record: smoking, chronic obstructive pulmonary disease and emphysema, tumour biology, histology and stage, neoadjuvant treatment, and adhesions or technical complexity. Those factors have paths to both the marker and the outcome which conditioning on sex does not close. A second group of unmeasured factors arises from the care episode rather than from the patient—chest-drain practice, discharge disposition, rehabilitation resource and surgeon preference—and has a path to the outcome alone. It follows that the attenuation of the association between C-reactive protein and prolonged stay when sex enters the model is consistent with confounding by sex and by factors associated with it, but does not by itself establish confounding in a causal sense while those common causes remain unmeasured. Everything below is an association in a single retrospective cohort.

Two things are true at once. Sex differences in circulating C-reactive protein are not new, having been documented in the general population and having motivated sex-specific reference intervals [26,27]; consistent with that, we found a sex difference in preoperative concentrations in both cohorts. What appears specific to lung resection is the amplification of that difference by the operation, together with a matching disadvantage in outcome. The baseline difference is general; the surgical response difference and the prognostic penalty are not.

Our findings extend the only closely comparable study, of 144 thoracic surgical patients, in which higher peaks with open surgery and more extensive resection were observed but did not reach significance and whose authors called for larger prospective work [16]. With five times the sample and an objective outcome, we add that the dominant determinant of the early concentration here is not the approach or the extent of resection but the sex of the patient. This is consistent with the established excess of pulmonary complications in men after lung resection, which has been ascribed to biological rather than case-mix factors [28], though without direct evidence of mechanism, and with sex differences in outcome after lobectomy, cardiac and hepatic surgery [33,34,35]; sex has also been used as a stratifying variable for bronchopleural fistula risk in a series of 4794 resections [57].

The comparison with the orthopaedic literature is informative rather than threatening. That the postoperative course of C-reactive protein is sex-specific after knee, hip and shoulder arthroplasty [17,18,19,20] means the phenomenon is not peculiar to this dataset, this institution or this assay; it replicates across operations sharing almost nothing except tissue injury, which argues that what is being measured is a property of the host response rather than of the operating list. What those studies did not do, and what we have done, is carry the observation to its consequence: if sex sets the level of the marker and also sets the outcome, the marker cannot be read as prognostic without adjustment. The negative control adds that the outcome half of that statement does not generalise across specialties, male sex carrying no penalty in general surgery at the same institution.

A sex difference in the magnitude of the acute-phase response is biologically plausible, and what follows is offered as speculation rather than as a demonstrated mechanism: sex hormones act on innate immune cells through oestrogen and androgen receptors and interact with glucocorticoid signalling [21,22], the lung shows sex-dependent immune behaviour in its own right [23,24], and human data during gender-affirming testosterone treatment show the innate compartment shifting with the hormonal environment [25]. We measured none of this, and cannot distinguish a hormonal mechanism from differences in body composition, smoking history, underlying pathology, or pleural manipulation not captured by the operation name; documented sex differences in chronic obstructive pulmonary disease and lung cancer biology would also be expected to contribute [36,58]. No hormonal, immunological, smoking, spirometric, or complication data were measured in this study, so every mechanistic statement in this paragraph is conjecture, and none of it is tested by our results.

The age analysis needs stating carefully, because it is the part of this report most open to over-interpretation. The inflammatory difference between the sexes was identical above and below 65 years; the outcome penalty was larger in the older group and reached conventional significance only there. It would be easy, and wrong, to present that as evidence that the effect grows with age. The formal interaction was null on every specification we tried, and the confidence interval in the younger stratum comfortably includes the older point estimate. The subgroup difference is compatible with chance.

What survives that caution is worth stating plainly. First, absolute risk: one older man in four stayed a week or longer, against one older woman in twelve, and prolonged stay was almost twice as common above 65 as below it. A relative effect of the same size produces far more excess days in the older group simply because the baseline is higher, which is the ordinary arithmetic of risk in geriatric populations and does not require an interaction to be clinically important. Second, the age-matched negative control: among older general surgical patients at the same institution, the odds ratio for male sex was 0.863, so the thoracic-specific pattern is not a general property of older surgical patients. Third, the direction of the age gradient across cut-offs is consistent, and the reduced physiological reserve that accompanies inflammageing offers a mechanism by which a fixed biological difference could produce a larger clinical one [6,7]. We regard the older patient as the population in which this finding matters, not as the population in which it has been shown to be stronger.

The practical implication concerns how the marker is used. C-reactive protein after thoracic surgery is interpreted against thresholds derived from mixed populations, and a failure to fall is treated as a signal warranting investigation [9,11,13]. If men run at about twice the concentration of women after the same operation, the same numerical threshold does not occupy the same position in the distribution for the two sexes. What follows from that for practice cannot be settled here. We performed no diagnostic-accuracy analysis, no calibration, no derivation of a cut-off, no external validation, and no formal assessment of clinical utility, and the outcome available to us is length of hospital stay rather than complications or infection. These data therefore do not derive, validate, or justify the implementation of sex-specific C-reactive protein thresholds, and we propose none. Age- and sex-dependent interpretation of C-reactive protein cut-offs has already been argued for in the general population [59], and sex-dependent reference intervals have been derived for other lung-related biomarkers [60]. That is the problem that led to sex-specific decision limits for cardiac troponin [38,39,61] and to the demonstration that sex-specific interpretation of preoperative haemoglobin alters perioperative risk estimates [40]. What our data show is narrower: the concentration reached on the first postoperative day differs systematically between the sexes in this cohort. Any study reporting postoperative C-reactive protein as a prognostic marker after thoracic surgery should state whether it was adjusted for sex, since the apparent association did not survive its inclusion here.

The choice of outcome matters. Treating critical care admission as physiological failure in this cohort would have attributed to inflammation what is a property of the operating list [49]. Length of stay is recorded for every patient, is what enhanced recovery pathways are designed to change, and is used as a quality metric after uncomplicated lobectomy [1,3], though it remains imperfect, influenced by discharge practice, social circumstances, and drain management as well as clinical events [4,5,62], and stay after video-assisted lobectomy varies measurably between surgeons within one institution [63]. In 13,099 lobectomies from which complications and deaths had been excluded, stay still spanned an interquartile range of 4 to 7 days and varied with payer, hospital volume and surgical approach [3]. Length of hospital stay is therefore an endpoint of the care episode and not a physiological measurement, and we refer to prolonged hospital stay throughout rather than to complications or to recovery.

What an elevated concentration in a man actually signifies is the first thing a clinician will want to know, and it cannot be settled from these data. The source dataset carries no complication coding, so we cannot say whether the prolonged stays in men reflect air leak, atelectasis, pneumonia, delayed drain removal, or none of these, and we cannot separate an inflammatory response that is larger but uneventful from one that is larger because something has gone wrong. The two available proxies are weak: critical care admission tracked the scheduled operation rather than deterioration, only 15 cases stayed in critical care for three days or longer, and in-hospital death occurred four times. This report therefore establishes a difference in a laboratory value and a difference in length of hospital stay; it does not establish that the first explains the second, nor that either reflects a difference in postoperative morbidity.

It follows that an isolated elevated concentration on the first postoperative day, in a man who is otherwise well, should not by itself start an investigation. In this cohort, the marker carried no information about prolonged stay once sex was in the model, in either sex and across thirteen model classes, and decision curve analysis found no threshold probability between 5% and 50% at which acting on it produced net benefit. The practical consequence is a caution rather than a test. The day 1 value is better read against the patient’s own preoperative concentration and against the change to day 3, which was the same in both sexes here, than against a fixed number; and where a fixed number is used, it should be read in the knowledge that men reach about twice the concentration of women after comparable operations. Clinical signs—fever, a rising oxygen requirement, prolonged air leak, and purulent drainage—remain the reason to investigate, and an isolated elevated concentration in a patient who is afebrile, eating and mobilising is not, on this evidence, a reason to delay discharge or to order imaging. This is an interpretive caution drawn from an association study, not a validated decision rule, and it should be treated as such.

The main strength is the internal negative control. Because the general surgical comparison comes from the same hospital, laboratory, and period, any explanation resting on assay calibration, on the Korean population, or on the era of practice would have to explain why it operates in one specialty and not the other; with 1479 events, the null result there is informative rather than underpowered.

Several limitations should be weighed, and the first is the most serious. Smoking raises circulating C-reactive protein chronically and worsens recovery after lung resection [64,65], and tobacco use in Korea remains far more common in men than in women [66]. Smoking could therefore raise both halves of our finding at once, and the source dataset does not release smoking status, so we cannot adjust for it. The negative control limits this explanation but does not dispose of it: patients undergoing lung resection are predominantly patients with lung cancer, a disease for which smoking is causal, so the male-to-female smoking gradient within the thoracic cohort is likely to be steeper than within general surgery, and a confounder stronger in one specialty than the other can produce exactly the contrast we report. The dataset does release an interpretation of preoperative spirometry and a preoperative diagnosis, and adding either to the models left both findings intact (Table 7); but an interpretive spirometry category is not a diagnosis of chronic obstructive pulmonary disease, and a preoperative diagnosis is not a pathological one. Emphysema, tumour histology and stage, neoadjuvant treatment, adhesions and technical complexity, and postoperative pulmonary complications are not released at all. Residual confounding by this group of factors is the principal threat to the validity of the present findings—not a secondary caveat—and it is why they are presented as associations and why the negative control is not treated as decisive.

This is also a single-centre retrospective analysis of a public dataset. The dataset carries no complication coding, so we cannot say whether the prolonged stays in men reflect pulmonary complications, air leak, or another cause: length of stay is what we could measure, not the mechanism. Day 3 C-reactive protein was available in only 54.4% of cases, and those are unlikely to be a random sample, a repeat measurement being more likely in patients causing concern; the exploratory trajectory analysis inherits that bias as well as its multiplicity problem. Open thoracotomy was used in only 46 cases, so the approach comparison that earlier work left unresolved cannot be examined here. Four patients died, and 15 stayed in critical care for three days or more, so hard outcomes could not be analysed. Two cases carried an implausible physical status grade and were retained. The source data were collected between August 2016 and June 2017, so the practice they describe is that of one institution in that period [45].

One further question raised in review, which factors decide the oncological outcome when segmentectomy is weighed against lobectomy in stage I non-small-cell lung carcinoma, lies outside what this dataset can address: no pathological diagnosis, stage, nodal status, resection margin, or recurrence information is released, and our outcome is length of hospital stay. The determinants established by the two randomised trials are radiological and pathological—a tumour 2 cm or less in diameter, a consolidation-to-tumour ratio above 0.5, peripheral location, and pathologically confirmed node-negative disease at hilar and mediastinal stations with an adequate margin. Under those conditions, segmentectomy was non-inferior, and for overall survival superior, to lobectomy in JCOG0802/WJOG4607L, and sublobar resection was non-inferior to lobar resection for disease-free survival in CALGB 140503 [67,68]. What our data can add is confined to the perioperative side of that decision. Among the 379 operations that were a lobectomy or a segmentectomy, the choice between them made no material difference to the early inflammatory response, an adjusted ratio of 1.132 (0.959 to 1.336, *p* = 0.143) for segmentectomy against lobectomy, whereas the difference between the sexes within those same 379 operations was 2.004 (1.761 to 2.280, *p* = 5.3 × 10^−23^). The sex of the patient moved the day 1 concentration considerably more than the extent of the resection did. That is a statement about a biomarker and not about oncological adequacy; the choice of operation should continue to rest on the criteria those trials established.

Confirmation should come from a multicentre cohort with C-reactive protein measured on fixed postoperative days, complication coding, smoking history, spirometric values rather than an interpretive category, pathological stage, and prespecified analysis by sex. Whether sex-specific thresholds improve on a single threshold can only be answered by deriving them in one cohort against a morbidity endpoint, calibrating them, and testing them in another; none of that is attempted here.

## 5. Conclusions

After lung resection, men mount an early inflammatory response roughly twice the size of that in women undergoing comparable operations, and among patients aged 65 years and older, more than one man in four stays in the hospital for a week or longer, compared with one woman in twelve. Neither pattern is present in older general surgical patients at the same institution. In this dataset and with the measured covariates, C-reactive protein on the first postoperative day was not independently associated with prolonged hospital stay after adjustment for sex and the other available factors; with 113 events the confidence interval for that estimate remains compatible with a modest association in either direction, and absence of prognostic value is not established. These data do not derive, validate, or justify sex-specific C-reactive protein thresholds and none is proposed: no diagnostic-accuracy, calibration, threshold-derivation, external-validation or clinical-utility analysis was performed, and the outcome measured is length of hospital stay rather than postoperative morbidity. Residual confounding by smoking, chronic airway disease, and tumour factors that this dataset does not record remains the principal alternative explanation for both findings. Whether the effect is genuinely larger in older patients than in younger ones is not established by these data; but the statement that the absolute risk is concentrated in them is not in doubt.

## Figures and Tables

**Table 1:** Baseline characteristics of the lung-resection cohort, overall and by sex.

Characteristic	All (*n* = 724)	Men (*n* = 381)	Women (*n* = 343)	*p*
Demographic				
Age, years	62 (55–70)	65 (57–72)	61 (53–69)	6.0 × 10^−6^
Age ≥ 65 years	313 (43.2)	192 (50.4)	121 (35.3)	4.1 × 10^−5^
Body mass index, kg/m^2^	23.4 (21.2–25.5)	23.6 (21.5–25.5)	23.1 (21.1–25.4)	0.112
Preoperative status				
ASA physical status ≥III [missing 20]	70 (9.9)	53 (14.3)	17 (5.1)	4.8 × 10^−5^
Hypertension	241 (33.3)	146 (38.3)	95 (27.7)	0.0025
Diabetes mellitus	84 (11.6)	49 (12.9)	35 (10.2)	0.265
C-reactive protein, mg/L	0.70 (0.30–2.50)	1.20 (0.40–3.60)	0.60 (0.20–1.60)	2.5 × 10^−8^
Albumin, g/L [missing 1]	42.0 (40.0–44.0)	42.0 (39.0–44.0)	42.0 (41.0–44.0)	0.010
White cell count, ×10^9^/L [missing 2]	6.06 (5.04–7.15)	6.25 (5.27–7.31)	5.79 (4.79–6.88)	7.6 × 10^−4^
Operative				
Major resection (lobectomy, bilobectomy, sleeve lobectomy or pneumonectomy)	347 (47.9)	200 (52.5)	147 (42.9)	0.0096
Minor resection: segmentectomy	62 (8.6)	31 (8.1)	31 (9.0)	0.665
Minor resection: wedge resection	217 (30.0)	101 (26.5)	116 (33.8)	0.032
Minor resection: metastasectomy	98 (13.5)	49 (12.9)	49 (14.3)	0.576
Open thoracotomy	46 (6.4)	38 (10.0)	8 (2.3)	2.6 × 10^−5^
Emergency operation	13 (1.8)	7 (1.8)	6 (1.7)	0.929
Duration of surgery, h	2.07 (1.58–2.70)	2.25 (1.80–3.08)	1.83 (1.50–2.31)	1.2 × 10^−11^
Early postoperative inflammatory response				
CRP on POD 1, mg/L	35.3 (20.2–54.2)	48.5 (34.1–67.9)	22.5 (14.7–34.8)	2.6 × 10^−48^
CRP on POD 3, mg/L [missing 330]	87.3 (51.5–138.2)	106.3 (73.5–164.6)	53.0 (31.7–87.6)	5.4 × 10^−20^
White cell count on POD 1, ×10^9^/L	10.86 (9.06–13.00)	10.72 (8.88–12.76)	11.07 (9.23–13.34)	0.046
Outcomes				
Postoperative stay, days	3.6 (2.4–5.5)	4.4 (2.6–6.5)	3.5 (2.3–4.5)	3.3 × 10^−9^
Postoperative stay ≥ 7 days	113 (15.6)	88 (23.1)	25 (7.3)	4.9 × 10^−9^
Postoperative stay ≥ 10 days	56 (7.7)	48 (12.6)	8 (2.3)	2.4 × 10^−7^
Any critical care admission	400 (55.2)	230 (60.4)	170 (49.6)	0.0035
Critical care stay ≥ 3 days	15 (2.1)	11 (2.9)	4 (1.2)	0.105
In-hospital death	4 (0.6)	4 (1.0)	0 (0.0)	0.126 (Fisher)

Values are median (interquartile range) or *n* (%). *p* values compare men with women: Mann–Whitney U test for continuous variables, chi-squared test for categorical variables, or Fisher’s exact test marked (Fisher). ASA physical status was missing in 20 cases and percentages for ASA are of cases with a recorded grade; two cases carried a recorded grade of VI and were retained as recorded. C-reactive protein on postoperative day 3 was available in 394 of 724 cases (54.4%). One case was performed by a robotic approach and is included in the cohort but not tabulated separately. ASA, American Society of Anesthesiologists; POD, postoperative day.

**Table 2:** Sex, early postoperative C-reactive protein, and prolonged postoperative stay.

Model	Cases	Events	Estimate (95% CI)	*p*
(**A**). Early postoperative CRP by sex (ratio of geometric means, men vs. women)				
CRP on POD 1, unadjusted	724		2.140 (1.924 to 2.379)	7.3 × 10^−40^
CRP on POD 1, adjusted	724		1.838 (1.656 to 2.040)	5.3 × 10^−28^
CRP on POD 3, adjusted (exploratory; 394 cases with POD 3 CRP)	394		1.798 (1.580 to 2.046)	1.7 × 10^−17^
(**B**). Male sex and postoperative stay ≥ 7 days (odds ratio, men vs. women)				
Unadjusted	724	113	3.820 (2.384 to 6.123)	2.6 × 10^−8^
Adjusted	724	113	2.529 (1.503 to 4.254)	4.7 × 10^−4^
Adjusted, with POD 1 CRP added to the model	724	113	2.874 (1.644 to 5.026)	2.1 × 10^−4^
(**C**). CRP on POD 1 and postoperative stay ≥ 7 days (odds ratio per doubling)				
Unadjusted	724	113	1.594 (1.292 to 1.967)	1.3 × 10^−5^
Adjusted, sex not in the model	724	113	1.037 (0.824 to 1.306)	0.754
Adjusted, sex in the model	724	113	0.865 (0.691 to 1.084)	0.208
Men only	381	88	0.867 (0.653 to 1.151)	0.324
Women only	343	25	0.873 (0.594 to 1.283)	0.490
(**D**). Negative control: general surgery at the same institution, same period				
CRP on POD 1 by sex, adjusted (ratio of geometric means)	1358		1.178 (1.002 to 1.385)	0.048
Male sex and stay ≥ 7 days, adjusted (odds ratio)	4930	1479	1.131 (0.952 to 1.344)	0.161
(**E**). Exploratory: CRP trajectory and postoperative stay ≥ 7 days (sex in the model)				
POD 3:POD 1 CRP ratio, per doubling, adjusted	394	89	1.463 (1.100 to 1.945)	0.0088
CRP on POD 3, per doubling, adjusted	394	89	1.134 (0.824 to 1.560)	0.441

Estimates in (**A**), and in the first row of (**D**), are ratios of geometric means obtained from linear regression of log2-transformed C-reactive protein, and are read as the multiple by which concentrations in men exceed those in women. All other estimates are odds ratios from logistic regression. Adjusted models include age (per 10 years), body mass index, ASA physical status III or higher with an indicator for a missing grade, emergency operation, extent of resection, open approach and duration of surgery; the general-surgery model replaces extent of resection with the recorded operation category. The primary outcome is a postoperative stay of 7 days or longer, which occurred in 113 of 724 cases (15.6%). (**e**) is exploratory: these models were fitted after several exposure and timepoint combinations had been examined, no correction for multiple comparisons has been applied, and the trajectory estimate should not be read as a confirmatory finding. CI, confidence interval; CRP, C-reactive protein; POD, postoperative day.

**Table 3:** Sequential adjustment of the association between C-reactive protein on postoperative day 1 and a stay of 7 days or longer.

Model	Variables in the Model	CRP on POD 1, aOR per Doubling (95% CI)	*p*	Male Sex, aOR (95% CI)	*p*
1	CRP on POD 1 alone (unadjusted)	1.594 (1.292 to 1.967)	1.3 × 10^−5^	not in model	
2	+age	1.502 (1.214 to 1.859)	1.8 × 10^−4^	not in model	
3	+sex and body mass index	1.195 (0.949 to 1.505)	0.129	3.062 (1.793 to 5.227)	4.1 × 10^−5^
4	+ASA ≥ III and emergency operation	1.155 (0.913 to 1.460)	0.230	2.996 (1.748 to 5.134)	6.6 × 10^−5^
5	+extent of resection and open approach	1.085 (0.857 to 1.372)	0.498	2.867 (1.666 to 4.931)	1.4 × 10^−4^
6	+duration of surgery (full model)	0.865 (0.691 to 1.084)	0.208	2.874 (1.644 to 5.026)	2.1 × 10^−4^
	Isolating the sex term (model 2 plus one covariate)				
2a	+body mass index only	1.523 (1.225 to 1.894)	1.5 × 10^−4^	not in model	
2b	+sex only	1.185 (0.944 to 1.486)	0.143	2.923 (1.722 to 4.959)	7.0 × 10^−5^

All models are logistic regressions fitted to the same 724 cases with 113 events, so estimates are directly comparable across rows, and each model adds the variables named to those of the model above. The association between C-reactive protein on postoperative day 1 and prolonged stay falls sharply when sex enters the model at step 3—the excess odds per doubling fall from 59% to 20% and the association is no longer statistically significant—and it is not recovered by any later covariate, whereas the estimate for male sex is essentially unchanged from step 3 onwards. Models 2a and 2b separate the two variables introduced at step 3: body mass index alone leaves the C-reactive protein estimate intact, whereas sex alone accounts for the whole of the change. aOR, adjusted odds ratio.

**Table 4:** Sex, early C-reactive protein and prolonged stay by age group and by specialty.

Group	Cases	Events	CRP Day 1, Men vs. Women (95% CI)	*p*	Male Sex, Odds Ratio (95% CI)	*p*
Lung resection						
Aged 65 years or older	313	65	1.825 (1.593 to 2.090)	1.8 × 10^−16^	3.031 (1.425 to 6.446)	0.0040
Aged under 65 years	411	48	1.873 (1.605 to 2.184)	1.2 × 10^−14^	1.905 (0.943 to 3.850)	0.072
All ages	724	113	1.838 (1.656 to 2.040)	5.3 × 10^−28^	2.529 (1.503 to 4.254)	4.7 × 10^−4^
General surgery, same institution						
Aged 65 years or older	1635	583	1.238 (0.917 to 1.672)	0.163	0.863 (0.654 to 1.141)	0.302
All ages	4930	1479	1.178 (1.002 to 1.385)	0.048	1.131 (0.952 to 1.344)	0.161

Estimates in the fourth column are adjusted ratios of geometric means for C-reactive protein on postoperative day 1 in men relative to women; those in the sixth are adjusted odds ratios for a postoperative stay of 7 days or longer in men relative to women. Lung-resection models in the two age strata use a reduced covariate set (age, physical status, extent of resection, open approach and duration of surgery) so that the ratio of events to parameters remains above ten; the all-ages row and the general-surgery rows use the full covariate set described in Table 2. The difference between the two age strata was tested formally and was not significant (ratio of odds ratios 1.403, 95% CI 0.501 to 3.929, *p* = 0.519 for age dichotomised at 65 years; 1.188 per decade, 0.768 to 1.837, *p* = 0.439 for age as a continuous variable).

**Table 5:** Sensitivity analyses of the two principal estimates.

Analysis	Cases	Events	CRP Day 1, Men vs. Women (95% CI)	*p*	Male Sex, Odds Ratio (95% CI)	*p*
Primary analysis (all 724 operations)	724	113	1.838 (1.656–2.040)	5.3 × 10^−28^	2.529 (1.503–4.254)	4.7 × 10^−4^
One operation per patient (716 operations)	716	111	1.851 (1.667–2.055)	2.3 × 10^−28^	2.634 (1.555–4.460)	3.1 × 10^−4^
All 724 operations, cluster-robust standard errors	724	113	1.838 (1.649–2.049)	4.2 × 10^−28^	2.529 (1.479–4.324)	7.0 × 10^−4^
All 724 operations, generalised estimating equations	724	113	1.864 (1.671–2.080)	6.4 × 10^−29^	2.538 (1.490–4.324)	6.1 × 10^−4^
Outcome: stay ≥10 days	724	56	1.838 (1.656–2.040)	5.3 × 10^−28^	4.457 (1.965–10.111)	3.5 × 10^−4^
Complete cases for physical status	704	113	1.810 (1.629–2.012)	3.9 × 10^−26^	2.529 (1.503–4.254)	4.7 × 10^−4^
Excluding two grade VI cases	722	112	1.837 (1.655–2.039)	5.8 × 10^−28^	2.544 (1.512–4.281)	4.4 × 10^−4^
Adding preoperative CRP to the model	724	113	1.741 (1.574–1.926)	3.6 × 10^−25^	2.450 (1.451–4.137)	8.0 × 10^−4^

Each row repeats the two principal analyses under a different definition of the cohort, outcome or model. Estimates in the fourth column are ratios of geometric means for C-reactive protein on postoperative day 1 in men relative to women; those in the sixth are odds ratios for the outcome in men relative to women. Adjustment is as in Table 2 throughout, except that the complete-case row omits the indicator for a missing physical status grade and the final row adds the preoperative C-reactive protein concentration. The second row retains one operation per patient, selected as the operation with the lower case identifier; case identifiers in the source dataset are random numbers and carry no temporal information, so this is one operation per patient chosen at random with respect to time and not the first operation in time. The third and fourth rows use all 724 operations while allowing for clustering within the 716 patients, by cluster-robust standard errors and by generalised estimating equations with an exchangeable working correlation respectively. The missing-grade indicator was perfectly predictive of a non-prolonged stay, none of the 20 cases with a missing grade having stayed 7 days or longer, so the complete-case row reproduces the primary estimate exactly.

**Table 6:** Sex and early C-reactive protein by surgical approach.

Subgroup	Cases	Men/Women	Events	CRP Day 1, Men vs. Women (95% CI)	*p*	Male Sex, Odds Ratio (95% CI)	*p*
All operations	724	381/343	113	1.838 (1.656–2.040)	5.3 × 10^−28^	2.529 (1.503–4.254)	4.7 × 10^−4^
Videoscopic (VATS) only	677	342/335	94	1.820 (1.634–2.026)	1.1 × 10^−25^	2.834 (1.643–4.886)	1.8 × 10^−4^
Open thoracotomy only	46	38/8	19	1.603 (0.985–2.608)	0.057	Not estimable	-
Videoscopic (VATS), aged 65 years or older	290	171/119	54	1.823 (1.588–2.093)	7.1 × 10^−16^	3.720 (1.636–8.458)	0.0017

Estimates in the fifth column are adjusted ratios of geometric means for C-reactive protein on postoperative day 1 in men relative to women; those in the seventh are adjusted odds ratios for a postoperative stay of 7 days or longer in men relative to women. Adjustment within a single approach omits the indicator for open thoracotomy and is otherwise as in Table 2. The odds ratio is not estimable in the 46 open operations, in which only eight patients were women. The formal interaction between sex and approach was null (ratio 0.931, *p* = 0.793). The one robotic operation is counted in the first row only. VATS, video-assisted thoracoscopic surgery.

**Table 7:** Sensitivity of the two principal estimates to recorded airway obstruction and to the indication for resection.

Model	CRP Day 1, Men vs. Women (95% CI)	*p*	Male Sex, Odds Ratio (95% CI)	*p*	CRP Day 1 per Doubling, Sex in the Model (95% CI)	*p*
Primary adjusted model	1.838 (1.656–2.040)	5.3 × 10^−28^	2.529 (1.503–4.254)	4.7 × 10^−4^	0.865 (0.691–1.084)	0.208
Adding obstructive spirometry pattern	1.876 (1.689–2.085)	3.5 × 10^−29^	2.558 (1.513–4.324)	4.5 × 10^−4^	0.861 (0.686–1.080)	0.195
Adding the full spirometry category	1.882 (1.693–2.091)	2.7 × 10^−29^	2.604 (1.533–4.423)	4.0 × 10^−4^	0.845 (0.672–1.061)	0.147
Adding indication for resection	1.839 (1.655–2.044)	1.5 × 10^−27^	2.591 (1.527–4.397)	4.2 × 10^−4^	0.866 (0.690–1.086)	0.213
Adding both	1.872 (1.683–2.083)	1.8 × 10^−28^	2.621 (1.537–4.469)	4.0 × 10^−4^	0.862 (0.686–1.083)	0.202

Each row repeats the two principal analyses and the association between C-reactive protein and prolonged stay after adding the stated variable to the adjusted model of Table 2. The obstructive spirometry pattern groups the mild, moderate, borderline and mixed obstructive categories of the recorded preoperative pulmonary function interpretation against all others; the full category enters all eight recorded levels. The indication is classified from the recorded preoperative diagnosis into primary lung cancer, metastasis, nodule or imaging abnormality, and other or benign. Neither variable is a substitute for smoking history, for a formal diagnosis of chronic obstructive pulmonary disease, or for pathological stage, none of which the source dataset releases.

**Table 8:** Absolute concentrations and the day 3 to day 1 ratio, by sex, in the 394 operations with both measurements.

Measure	Men	Women	Adjusted Ratio, Men vs. Women (95% CI)	*p*	Odds Ratio per Doubling for Stay ≥7 Days, Sex in the Model (95% CI)	*p*
CRP on postoperative day 1 (mg/L)	52.4	25.7	1.824 (1.628–2.044)	1.9 × 10^−22^	0.643 (0.451–0.916)	0.015
CRP on postoperative day 3 (mg/L)	104.2	50.9	1.798 (1.580–2.046)	1.7 × 10^−17^	1.134 (0.824–1.560)	0.441
Day 3:day 1 ratio	1.99	1.98	0.985 (0.855–1.136)	0.839	1.463 (1.100–1.945)	0.0088

Values for men and women are geometric means; the first two rows are in mg/L and the third is a dimensionless ratio. The fourth column gives the adjusted ratio of geometric means in men relative to women, and the sixth the adjusted odds ratio for a postoperative stay of 7 days or longer per doubling of the measure, with sex in the model. Adjustment is as in Table 2. This table is exploratory and is restricted to the 394 operations with a day 3 measurement, which are a selected subgroup: a day 3 value was available in 65.9% of men against 41.7% of women, and in 78.8% of operations followed by a stay of 7 days or longer against 49.9% of the rest. The day 1 odds ratio within this subgroup is distorted by that selection and is not the primary estimate, which is given in Table 2.

## Data Availability

The source data are publicly available from VitalDB at https://vitaldb.net (accessed on 20 August 2026) under a Creative Commons Attribution-NonCommercial-ShareAlike 4.0 International licence; the clinical information and laboratory tables analysed here were retrieved on 20 August 2026 from https://api.vitaldb.net/cases and https://api.vitaldb.net/labs. All analysis code, the derived analytical cohort, the variable mapping from the source fields, the documented assignment of the postoperative time windows, the environment specification and the outputs behind every table and figure in the manuscript and the supplement are publicly available at https://github.com/sanghyunn82-creator/vitaldb-lung-resection-crp (accessed on 30 September 2026).

## Supplementary Materials

The following supporting information can be downloaded at: https://www.mdpi.com/article/10.3390/diseases14100369/s1. Figure S1, flow of surgical cases from the source dataset to the analytical cohort; Table S1, definition and source of each variable used in the analysis; Table S2, complete sensitivity analyses of the two principal estimates; Table S3, fit and calibration of the logistic regression models; Figure S2, out-of-fold receiver operating characteristic curves for logistic regression and random forest; Figure S3, permutation importance of each variable, expressed as the mean fall in cross-validated discrimination; Table S4, variance inflation factors for the covariates of the full model; Figure S4, cross-validated area under the ROC curve for thirteen model classes across four variable sets; Figure S5, structure of the relationship between sex, C-reactive protein and prolonged stay; Table S5, cross-validated discrimination for thirteen model classes and four variable sets; Table S6, structural analyses of the relationship between sex and C-reactive protein.
