# Supplementary material for: Male Sex, Early Postoperative C-Reactive Protein and Prolonged Hospital Stay After Lung Resection in Older Patients: A Retrospective Cohort Study
Source: Diseases. 2026 Oct 6;14(10):369. doi: 10.3390/diseases14100369 (PMC13703708; doi:10.3390/diseases14100369)
Supplement: Supplementary file 1 [file diseases-14-00369-s001.zip › diseases-4572820-supplementary.pdf]

Supplementary Materials

Male Sex, Early Postoperative C-Reactive Protein and Prolonged Hospital Stay after Lung Resection in Older Patients: A Retrospective Cohort Study

Yeon Soo Kim and So Hyun Ahn

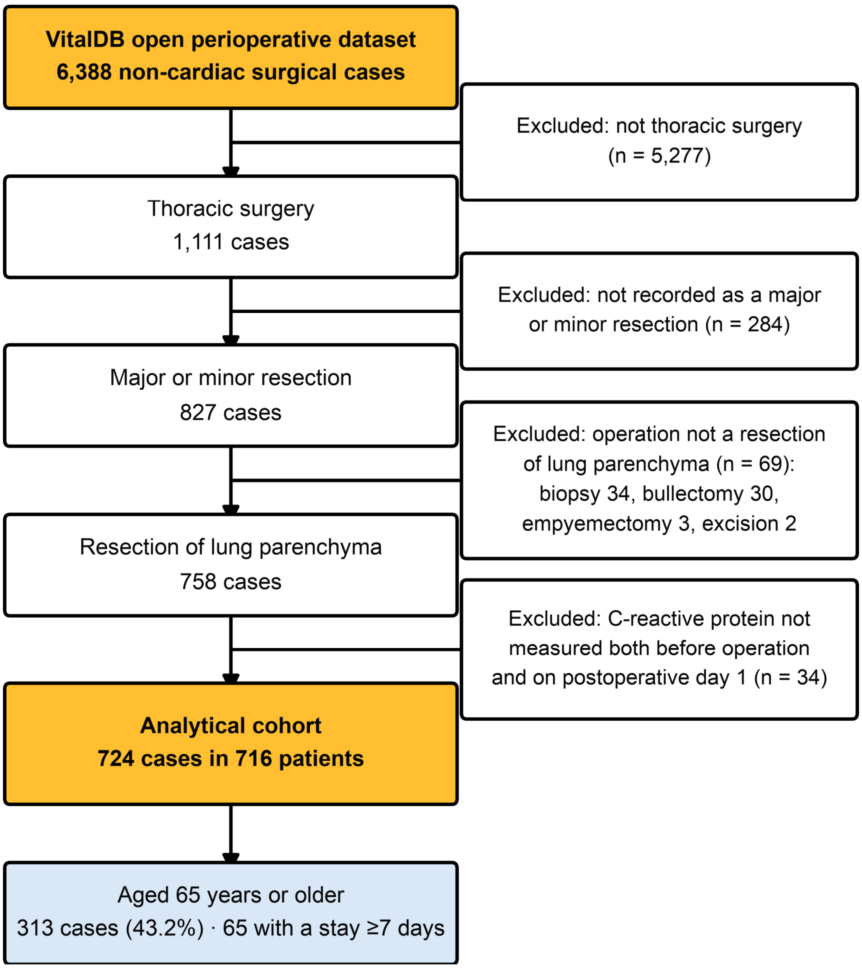

Figure S1. Flow of surgical cases from the source dataset to the analytical cohort. Percentages in the lowest box are of the 724 analytical cases.

Table S1. Definition and source of each variable used in the analysis.

Variable	Definition	Source field
Male sex	Sex as recorded in the dataset; men coded 1, women 0	cases.csv, sex
Age	Age at operation, in years; entered per 10 years	cases.csv, age
Body mass index	Weight divided by height squared, kg/m²	cases.csv, bmi
ASA physical status ≥III	American Society of Anesthesiologists grade III or above; a separate indicator flags the 20 cases with no recorded grade	cases.csv, asa
Emergency operation	Operation recorded as emergency	cases.csv, emop
Major resection	Operation type recorded as major resection (lobectomy, bilobectomy, sleeve lobectomy or pneumonectomy) versus minor	cases.csv, optype
Open thoracotomy	Surgical approach recorded as open, versus videoscopic or robotic	cases.csv, approach
Duration of surgery	End minus start of operation, in hours	cases.csv, opstart/opend

Preoperative CRP	Last C-reactive protein within 30 days before the start of the case, mg/L	labs.csv, name = crp, dt < 0
CRP on postoperative day 1	Last result between the end of operation and 36 hours afterwards, mg/L	labs.csv, name = crp, 0 < day ≤ 1.5
CRP on postoperative day 3	Last result between 60 and 96 hours after operation, mg/L	labs.csv, name = crp, 2.5 < day ≤ 4
Postoperative stay	Discharge minus end of operation, in days	cases.csv, opend/dis
Primary outcome	Postoperative stay of 7 days or longer	derived
Critical care admission	Any recorded postoperative critical care stay; not used as an outcome (see Methods)	cases.csv, icu_days

All fields are taken from the public VitalDB release. C-reactive protein is reported in mg/L; the dataset records it in mg/dL and values were multiplied by ten.

Table S2. Complete sensitivity analyses of the two principal estimates.

**Analysis**	**Cases**	**Events**	**CRP day 1, men vs women (95% CI)**	**p**	**Male sex, odds ratio (95% CI)**	**p**
Primary analysis, all ages	724	113	1.838 (1.656 to 2.040)	5.3×10^{-28}	2.529 (1.503 to 4.254)	4.7×10^{-4}
Aged ≥60 years	452	84	1.798 (1.592 to 2.032)	2.0×10^{-19}	2.831 (1.509 to 5.313)	0.0012
Aged ≥65 years	313	65	1.825 (1.593 to 2.090)	1.8×10^{-16}	3.031 (1.425 to 6.446)	0.0040
Aged ≥70 years	201	44	1.766 (1.481 to 2.105)	1.3×10^{-9}	3.746 (1.454 to 9.650)	0.0062
Aged <65 years	411	48	1.873 (1.605 to 2.184)	1.2×10^{-14}	1.905 (0.943 to 3.850)	0.072
Outcome: stay ≥10 days, all ages	724	56	1.838 (1.656 to 2.040)	5.3×10^{-28}	4.457 (1.965 to 10.111)	3.5×10^{-4}
Complete cases for ASA grade	704	113	1.810 (1.629 to 2.012)	3.9×10^{-26}	2.529 (1.503 to 4.254)	4.7×10^{-4}
Excluding two ASA grade VI cases	722	112	1.837 (1.655 to 2.039)	5.8×10^{-28}	2.544 (1.512 to 4.281)	4.4×10^{-4}
One case per patient	716	111	1.851 (1.667 to 2.055)	2.3×10^{-28}	2.634 (1.555 to 4.460)	3.1×10^{-4}
Adding preoperative CRP	724	113	1.741 (1.574 to 1.926)	3.6×10^{-25}	2.450 (1.451 to 4.137)	8.0×10^{-4}

Ratios of geometric means for C-reactive protein use the full covariate set throughout; odds ratios in the age strata use the reduced covariate set described in the Methods. The row for a stay of 10 days or longer changes only the outcome, so the C-reactive protein estimate is unchanged from the primary analysis.

Table S3. Fit and calibration of the logistic regression models.

**Model**	**Cases**	**Events**	**Parame-ters**	**Events per parameter**	**Area under the ROC curve**	**Hosmer–Lemeshow χ^2**	**p**	**Log-likeli-hood**	**AIC**
Whole cohort, full model	724	113	9	12.6	0.802	7.01	0.536	-251.0	522.0
Aged ≥65 years, reduced model	313	65	6	10.8	0.768	10.83	0.212	-136.1	286.2
Aged <65 years, reduced model	411	48	6	8.0	0.755	13.40	0.099	-123.2	260.5

A Hosmer–Lemeshow p value above 0.05 indicates no evidence of poor calibration. The reduced model in patients aged under 65 years has 8.0 events per parameter, below the conventional minimum of ten; estimates from that stratum should be read with that in mind.

Supplementary Methods. Model classes and structural analyses

The thirteen model classes were logistic regression with and without elastic-net penalisation, a support vector machine with a radial basis kernel, k-nearest neighbours, naive Bayes, a random forest, extremely randomised trees, AdaBoost, gradient boosting, histogram-based gradient boosting, XGBoost, LightGBM, and a fully connected neural network with two hidden layers of 64 and 32 units and dropout of 0.35. These were fitted with scikit-learn 1.9.0, XGBoost 3.2.0, LightGBM 4.7.0 and PyTorch 2.4.1 on a single graphics processing unit. Each class was fitted to four variable sets — the clinical covariates alone, and with C-reactive protein, with sex, and with both — and discrimination was estimated by repeated stratified five-fold cross-validation with five repetitions, giving 25 fits per combination.

Permutation importance is the mean fall in the area under the curve over 15 random shuffles of each variable; Shapley additive explanation values and their pairwise interaction terms were computed for the full variable set. Four further analyses examined the structure of the relationship rather than its prediction: the proportion of the variance in day 1 C-reactive protein attributable to sex over and above the clinical covariates, estimated as a partial coefficient of determination; a sex-by-marker interaction term in the primary model and in cross-validation; models fitted separately within each sex; and decision curve analysis, which expresses the net benefit of a model across the range of threshold probabilities at which a clinician might act. These analyses address model form and structure, not causation, and are reported as checks on the primary models rather than as alternatives to them.

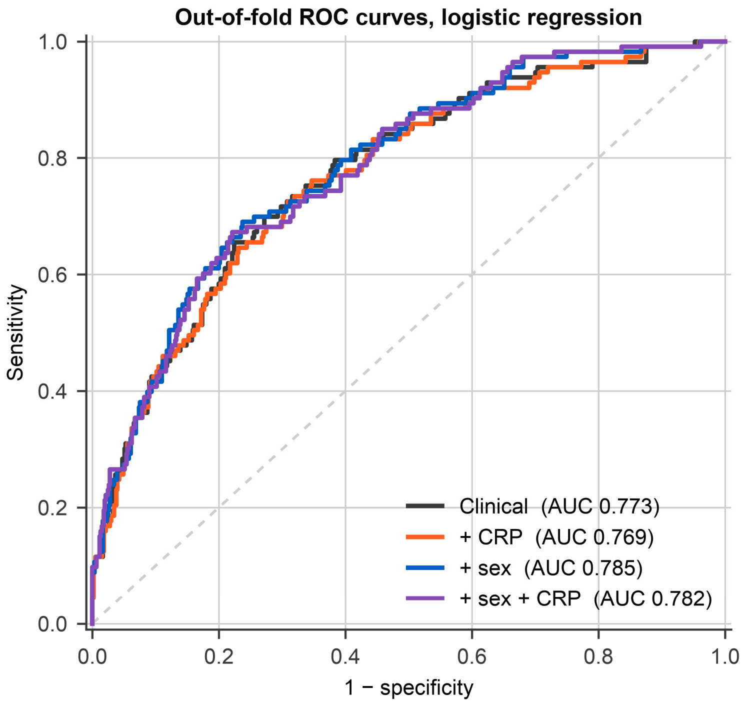

Figure S2. Out-of-fold receiver operating characteristic curves for logistic regression fitted to four variable sets. Curves are pooled across a single stratified five-fold split of the 724 cases. Adding C-reactive protein to the clinical covariates does not shift the curve; adding sex does.

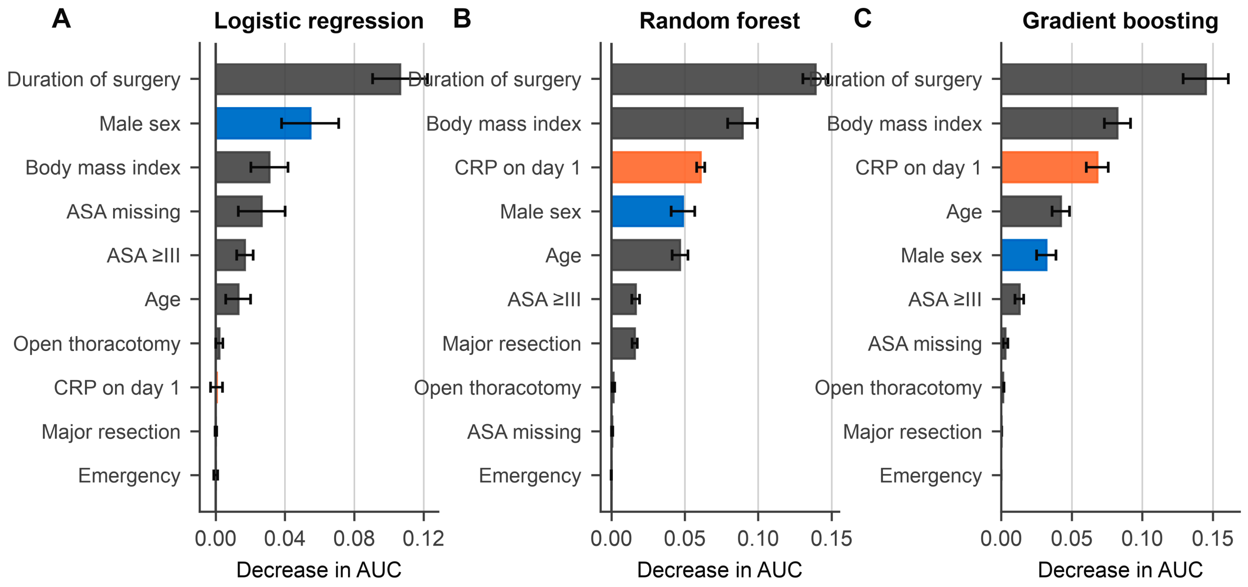

Figure S3. Permutation importance of each variable, expressed as the mean fall in the area under the ROC curve over 15 random shuffles, with one standard deviation. Panel A, logistic regression; panel B, random forest; panel C, gradient boosting. Male sex is shown in blue and C-reactive protein on day 1 in orange. In the two tree-based models the marker ranks above sex; this reflects the overlap between them, since a flexible model can use the marker as a proxy for sex. Permutation importance describes what a fitted model relies on, not what determines the outcome, and should be read alongside the cross-validated comparison in Figure 5 of the main text.

Table S4. Variance inflation factors for the covariates of the full model.

**Covariate**	**Variance inflation factor**
Male sex	1.09
Age (per 10 years)	1.09
Body mass index	1.01
ASA physical status ≥III	1.08
Emergency operation	1.01
Major resection	1.12
Open thoracotomy	1.16
Duration of surgery (h)	1.25

All values are below 1.3, indicating no material collinearity among the covariates.

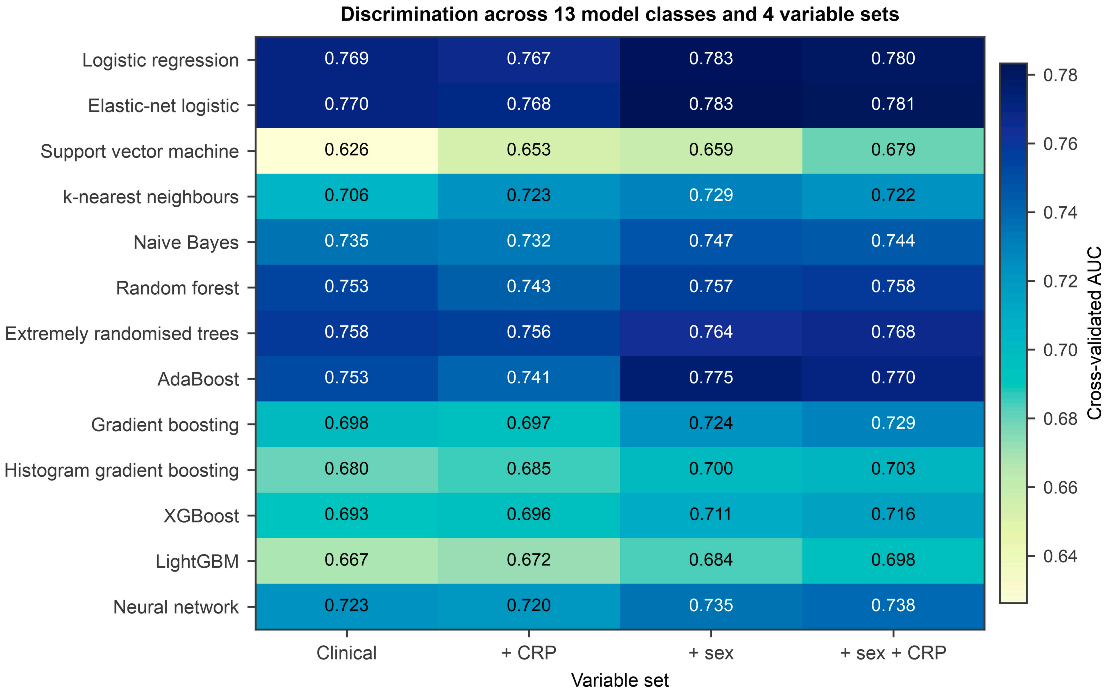

Figure S4. Cross-validated area under the ROC curve for thirteen model classes across four variable sets, from repeated stratified five-fold cross-validation with five repetitions. Darker cells indicate better discrimination.

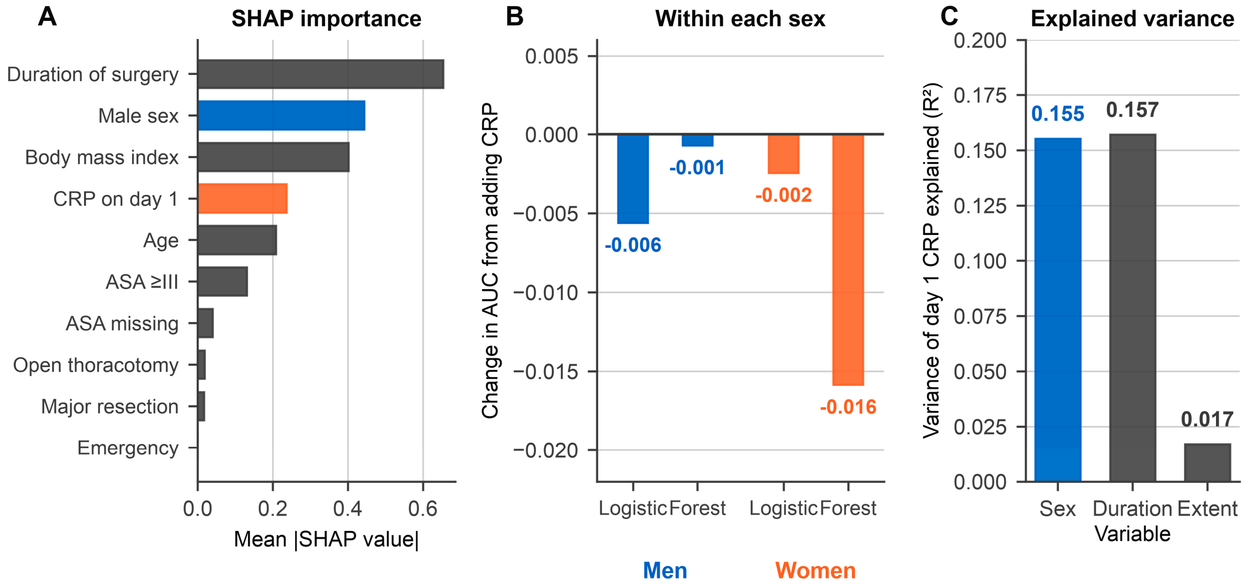

Figure S5. Structure of the relationship between sex, C-reactive protein and prolonged stay. Panel A, mean absolute Shapley value for each variable in a gradient boosting model; male sex is shown in blue and C-reactive protein in orange. Panel B, the change in cross-validated area under the ROC curve produced by adding C-reactive protein within each sex separately, for logistic regression and for a random forest. Panel C, the proportion of the variance in day 1 C-reactive protein explained by sex over and above the clinical covariates, with duration of surgery and extent of resection shown for comparison.

Table S5. Cross-validated discrimination for thirteen model classes and four variable sets.

Model class	Clinical	+ CRP	+ sex	+ sex + CRP	Δ from CRP	Δ from sex
Logistic regression	0.769 (0.046)	0.767 (0.045)	0.783 (0.044)	0.780 (0.043)	-0.0025	+0.0131
Elastic-net logistic	0.770 (0.045)	0.768 (0.044)	0.783 (0.043)	0.781 (0.043)	-0.0016	+0.0134
Support vector machine	0.626 (0.049)	0.653 (0.042)	0.659 (0.043)	0.679 (0.041)	+0.0270	+0.0323
k-nearest neighbours	0.706 (0.049)	0.723 (0.047)	0.729 (0.054)	0.722 (0.055)	+0.0166	+0.0230
Naive Bayes	0.735 (0.056)	0.732 (0.055)	0.747 (0.053)	0.744 (0.054)	-0.0031	+0.0116
Random forest	0.753 (0.047)	0.743 (0.045)	0.757 (0.045)	0.758 (0.043)	-0.0106	+0.0035
Extremely randomised trees	0.758 (0.053)	0.756 (0.051)	0.764 (0.049)	0.768 (0.048)	-0.0021	+0.0057
AdaBoost	0.753 (0.042)	0.741 (0.040)	0.775 (0.046)	0.770 (0.047)	-0.0111	+0.0227
Gradient boosting	0.698 (0.046)	0.697 (0.044)	0.724 (0.046)	0.729 (0.050)	-0.0015	+0.0259
Histogram gradient boosting	0.680 (0.050)	0.685 (0.045)	0.700 (0.049)	0.703 (0.047)	+0.0050	+0.0204
XGBoost	0.693 (0.053)	0.696 (0.048)	0.711 (0.050)	0.716 (0.047)	+0.0030	+0.0176
LightGBM	0.667 (0.052)	0.672 (0.044)	0.684 (0.047)	0.698 (0.046)	+0.0049	+0.0170
Neural network	0.723 (0.047)	0.720 (0.046)	0.735 (0.045)	0.738 (0.036)	-0.0028	+0.0118

Values are the mean area under the ROC curve with the standard deviation across 25 fits in parentheses. The final two columns give the change relative to the clinical covariates alone. Adding sex improved discrimination in all thirteen model classes; adding C-reactive protein improved it in five.

Table S6. Structural analyses of the relationship between sex and C-reactive protein.

Quantity	Value
Variance of day 1 CRP explained by clinical covariates (R^2)	0.190
Variance explained after adding sex (R^2)	0.316
Partial R^2 attributable to sex	0.155
Partial R^2 for duration of surgery, for comparison	0.157
Partial R^2 for extent of resection, for comparison	0.017
Sex × CRP interaction, ratio of odds ratios (95% CI)	0.940 (0.596 to 1.481)
Sex × CRP interaction, p value	0.789
Change in cross-validated AUC from adding the interaction term	-0.0020
Change in AUC from adding CRP, men only (logistic / random forest)	-0.0056 / -0.0007
Change in AUC from adding CRP, women only (logistic / random forest)	-0.0024 / -0.0159
DeLong p, clinical model versus clinical + CRP	0.098
DeLong p, clinical model versus clinical + sex	0.315
Mean |SHAP| for male sex (rank among 10 variables)	0.444 (2nd)
Mean |SHAP| for CRP on day 1 (rank among 10 variables)	0.236 (4th)
SHAP interaction strength, sex × CRP (rank among 45 pairs)	0.0153 (13th)

The partial coefficient of determination is the share of the variance in day 1 C-reactive protein not explained by the clinical covariates that is explained by sex. Shapley values were computed for a gradient boosting model fitted to all 724 cases.
